# Engineering Strategies in Microorganisms for the Enhanced Production of Squalene: Advances, Challenges and Opportunities

**DOI:** 10.3389/fbioe.2019.00050

**Published:** 2019-03-22

**Authors:** Nisarg Gohil, Gargi Bhattacharjee, Khushal Khambhati, Darren Braddick, Vijai Singh

**Affiliations:** ^1^School of Biological Sciences and Biotechnology, Institute of Advanced Research, Koba Institutional Area, Gandhinagar, India; ^2^Department of R&D, Cementic S. A. S., Genopole, Paris, France

**Keywords:** squalene, metabolic engineering, fermentation, biosynthesis, production, synthetic biology, anti-oxidant, anti-aging

## Abstract

The triterpene squalene is a natural compound that has demonstrated an extraordinary diversity of uses in pharmaceutical, nutraceutical, and personal care industries. Emboldened by this range of uses, novel applications that can gain profit from the benefits of squalene as an additive or supplement are expanding, resulting in its increasing demand. Ever since its discovery, the primary source has been the deep-sea shark liver, although recent declines in their populations and justified animal conservation and protection regulations have encouraged researchers to identify a novel route for squalene biosynthesis. This renewed scientific interest has profited from immense developments in synthetic biology, which now allows fine-tuning of a wider range of plants, fungi, and microorganisms for improved squalene production. There are numerous naturally squalene producing species and strains; although they generally do not make commercially viable yields as primary shark liver sources can deliver. The recent advances made toward improving squalene output from natural and engineered species have inspired this review. Accordingly, it will cover in-depth knowledge offered by the studies of the natural sources, and various engineering-based strategies that have been used to drive the improvements in the pathways toward large-scale production. The wide uses of squalene are also discussed, including the notable developments in anti-cancer applications and in augmenting influenza vaccines for greater efficacy.

## Introduction

Squalene (C_30_H_50_), an intermediate of cholesterol biosynthesis, is a naturally occurring highly unsaturated triterpenic hydrocarbon compound that forms solely via the mevalonic acid (MVA) or 2-C-methyl-D-erythritol 4-phosphate (MEP) pathways (Spanova and Daum, 2011; Popa et al., 2015; Rani et al., 2018). It was first described and identified in 1916 in deep-sea shark (*Squalus* spp.) liver by a Japanese chemist Mitsumaru Tsujimoto, hence the name “squalene” (Tsujimoto, 1916). Since its discovery, shark liver oil has been the largest source of squalene. A wide range of microorganisms including yeast such as *Saccharomyces cerevisiae* (Mantzouridou and Tsimidou, 2010) and *Torulaspora delbrueckii* (Bhattacharjee et al., 2001), fungus *Aurantiochytrium* sp. (Jiang et al., 2004; Nakazawa et al., 2014; Pora et al., 2014), *Euglena* (Anding et al., 1971), archaea *Halobacterium cutirubrum*, and several other species have the natural ability to produce squalene. Apart from sources of microbial origin, a number of reports have described the production of squalene from some plants, predominantly amaranth (Lyon and Becker, 1987; Czaplicki et al., 2011; Naziri et al., 2011b; Rosales-García et al., 2017b) and olive (Gutfinger and Letan, 1974; Frega et al., 1992; Nenadis and Tsimidou, 2002; Beltrán et al., 2016). As far as both plant and microbial sources are concerned, productivity still remains a major issue for scale-up, thereby, limiting their use as sources in industrial and biomedical applications (Spanova and Daum, 2011; Lozano-Grande et al., 2018).

In humans, squalene is one of the major components of sebum (12%), epidermis (<0.5%), and surface lipids (10%) (Nicolaides, 1974). It is synthesized by the liver and secreted largely from the sebaceous glands (Popa et al., 2015). The amount of secreted squalene ranges from 125 to 475 mg/day, depending upon the individuals and their diet (Nikkari et al., 1974). According to the clinical research data, about 60–85% of the total orally administrated squalene is absorbed and distributed efficiently to different tissues (Gabás-Rivera et al., 2014), namely the skin where it plays a salient role as an antioxidant to protect against the free radicals and environmental oxidative stress (Kohno et al., 1995). In most cases, after the age of 30, the concentration of squalene starts to decline (Popa et al., 2015). Therefore, it is preferable to supply exogenous squalene (about 500 mg/day) for maintaining a healthy lifestyle (Günes, 2013).

Squalene has been known to play diverse biological roles as an anti-oxidant (Amarowicz, 2009; Günes, 2013), anti-cancer agent (Kim and Karadeniz, 2012; Günes, 2013), age defyer (Huang et al., 2009; Popa et al., 2015), chemopreventive agent (Aioi et al., 1995; Budiyanto et al., 2000; Smith, 2000), anti-bacterial agent (Kopicová and Vavreinová, 2007; Popa et al., 2015), adjuvant for vaccines and drug carrier (Del Giudice et al., 2006; Pasquale et al., 2015), and detoxifier (Kim and Karadeniz, 2012; Ivanova et al., 2015) among others. Thus, it is a favored choice in pharmaceuticals, cosmetic industries, and food supplements (Popa et al., 2015; Lozano-Grande et al., 2018). The role of squalene is not just confined to these applications, but it is also a precursor to thousands of bioactive molecules, including steroids and hopanoids. Consequently, many chemical, food, cosmetic, and pharmaceutical industries have started to use squalene extensively. Over the last decade, global squalene demand has increased and gained much public and scientific attention. In 2014, the global squalene market demand was about 2.67 kilotons (Rosales-Garcia et al., 2017a), with a projected value of 241.9 million USD by 2022, with major revenues expected from the personal care and cosmetic products (Global Market Insights, 2016). In order to fulfill this ever-increasing demand of squalene, a pressing need has arisen to produce squalene in a renewable and sustainable manner.

Rapid and remarkable advances in genetic engineering, metabolic engineering and synthetic biology approaches over the last few decades have facilitated the insertion of heterologous genes and editing of the genome of an organism, enabling successful attempts to produce innumerable molecules of industrial importance (Martin et al., 2009; Stephanopoulos, 2012; Singh, 2014; Jullesson et al., 2015). Lately, a number of microorganisms have been engineered by inserting a squalene biosynthetic pathway or by the modification of existing biosynthetic pathway for over-production of squalene (Ghimire et al., 2009; Katabami et al., 2015;Han et al., 2018; Wei et al., 2018).

This review discusses the various sources of squalene, and therein the positive and negative aspects of their utilization toward large-scale production. Furthermore, we describe the native biosynthetic pathways which are present in many microorganisms and how these pathways can be transformed or extended to convert the organisms into “cellular factories” for squalene production. We summarize recently developed strategies that can overproduce this commercially valuable molecule. Through the review, we also point out possible strategies that might help to resolve the obstacles faced in squalene biosynthesis. Lastly, we highlight the ways to exploit the biological roles of squalene and its applications in manufacturing a wide range of industrially and medically important products.

## Natural Sources of Squalene

**Figure 1:**
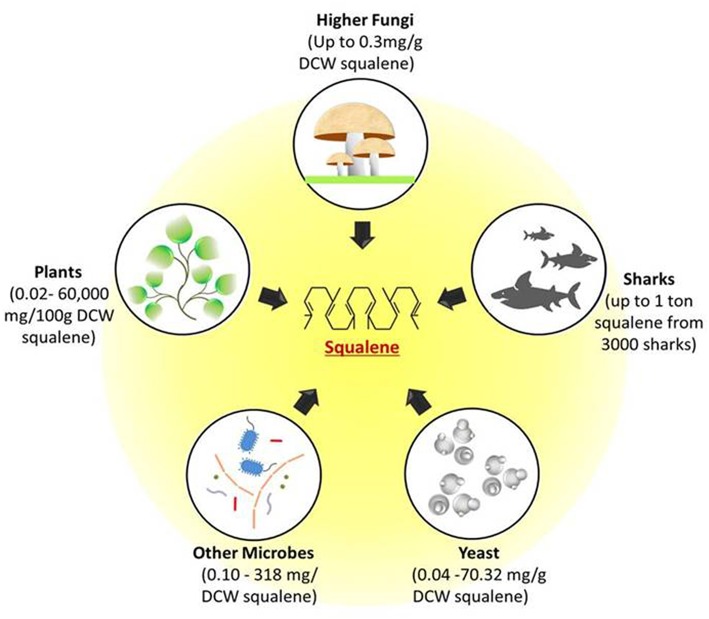
Potential natural sources of squalene. Figure depicts the possible squalene sources ranging from unicellular microbes such as yeast and other bacterial cells to multicellular fungi, plants, and deep sea sharks. All these sources can produce squalene through mevalonate pathway. DCW, Dry cell weight.

The importance of squalene with regards to its properties and applications has attracted many industries and research groups to discover rich sources of squalene in nature. The hunt for the novel sources is not just limited to its accessibility but also to the feasibility and ease of extraction. The accessible squalene sources such as yeast, fungi, plants, and deep sea sharks are shown in Figure 1.

### Squalene From Shark Liver Oil

Over the years, deep-sea shark liver oil has been the major natural source of squalene (Ronco and De Stéfani, 2013; Popa et al., 2015; Rosales-Garcia et al., 2017a). It is estimated that for producing 1 ton of squalene, it demands as many as about 3,000 sharks (Ciriminna et al., 2014). A single distillation under vacuum at a temperature of 200–230°C is required for recovering >98% purified squalene from the liver oil (Ciriminna et al., 2014). Every year, approximately 100 million sharks are killed brutally for the purpose of shark finning (Tsoi et al., 2016), with most of it fulfilling the global demand of squalene for cosmetic industries (Ciriminna et al., 2014).

The long reproductive cycle and slow growth rate of sharks in tandem with the reckless fishing have taken a grave toll on their numbers, which now threatens to reach near extinction. This rapid depletion of shark populations is taking place at a lightning speed, far exceeding its recovery. In an attempt to conserve shark populations, numerous shark breeding and importing countries such as the United States of America, Costa Rica, New Zealand, Taiwan, Australia, and those included in the European Union have banned shark killing and finning (Dulvy et al., 2008). Apart from that, considering the similarities that squalene share with the other lipid components present in shark liver oil, the process of its purification as a separate entity is cumbersome. Nowadays, oceans are heavily polluted with tons of trash, oil spills, fertilizers, pesticides, chemicals, plastics, heavy metals, organic and other persistent organic pollutants (POPs), which results from man-made ecocide (Seltenrich, 2015; Ramírez-García et al., 2018). These pollutants can still be recovered even after purification of squalene from the shark (Popa et al., 2014). Therefore, with the population and contamination concerns, it becomes an utmost necessity to pursue the extraction and production of squalene from improved and renewable sources.

### Squalene From Plants

Besides shark liver oil, most plant oils contain a minute amount of squalene (Czaplicki et al., 2012). This phytosqualene has an edge over the shark squalene, in terms that it is highly stable, non-toxic, odorless, and colorless (Popa et al., 2014), favoring its use in cosmetic and pharmaceutical industries. Additionally, the plant based squalene has the capability to reduce the risk of different types of cancer by minimizing serum cholesterol levels (He et al., 2003). Phytosqualene was first found and extracted from olive oil (Thorbjarnarson and Drummond, 1935), and since then, the quest for alternatives is still in progress. Interestingly, amongst the known plant sources, olive oil (0.9–12.45 g/kg squalene; Giacometti and Milin, 2001) is the only source used for commercial purposes (Lozano-Grande et al., 2018), despite the fact that amaranth has the highest squalene content (600 g/kg) of all the reported plant sources (Wejnerowska et al., 2013). The reason behind this is that the lipid content present in olives (6.67–26.67%) (United States Department of Agriculture, 2018a,b) is greater than the amaranth seeds (4.8–8.1%) (Saunders and Becker, 1984; United States Department of Agriculture, 2018c), although the amount recovered is still insufficient to meet the global demands.

Rice bran, a co-product of the rice milling process also contains a good amount (318.9–320 mg/100 g) of squalene (Rukmini and Raghuram, 1991; Pokkanta et al., 2019). Palm oil has just 20–50 mg/100 g of squalene (Goh et al., 1985; Lau et al., 2005) but because of its large-scale production, it can be considered as an acceptable source of the squalene overall. Apart from this, avocado (34–37 mg/100 g squalene) (Gutfinger and Letan, 1974) has also been reported to contain a meager amount of squalene. Some nuts also contain small amounts of squalene, including brazil nut (145.8 mg/100 g) (Derewiaka et al., 2014), peanut (27.4–132.9 mg/100 g) (Frega et al., 1992; Tuberoso et al., 2007; Pokkanta et al., 2019), hazelnut (9.3–39.2 mg/100 g) (Frega et al., 1992; Bada et al., 2004; Derewiaka et al., 2014), macadamia (7.2–38.3 mg/100 g) (Maguire et al., 2004; Wall, 2010; Derewiaka et al., 2014), pecan (20.8–29.8 mg/100 g) (Derewiaka et al., 2014; Fernandes et al., 2017), pistachio (5.5–22.6 mg/100 g) (Derewiaka et al., 2014; Salvo et al., 2017), cashew (11.6 mg/100 g) (Derewiaka et al., 2014), almond (1.3–9.6 mg/100 g) (Liu et al., 1976; Fernandes et al., 2017), and walnut (0.09–0.94 mg/100 g).

Seeds such as ginseng (514–569 mg/100 g) (Beveridge et al., 2002), soybean (3–22 mg/100 g) (Gutfinger and Letan, 1974; Frega et al., 1992; Maguire et al., 2004; Naziri et al., 2011b; Pokkanta et al., 2019), sunflower seed (0–19 mg/100 g) (Tuberoso et al., 2007; Naziri et al., 2011b), sesame seed (57.2–60.7 mg/100 g) (Pokkanta et al., 2019), coriander seed (45.1 mg/100 g) (Pokkanta et al., 2019), pumpkin seed (260–523 mg/100 g) (Tuberoso et al., 2007; Czaplicki et al., 2011; Naziri et al., 2011b), flaxseed (1.0–4.2 mg/100 g) (Tanska et al., 2016), rapeseed (43.7 mg/100 g) (Tuberoso et al., 2007), grape seed (10.2–16.2 mg/100 g) (Frega et al., 1992; Wen et al., 2016), cottonseed (2.7–9.1 mg/100 g) (Gutfinger and Letan, 1974; Liu et al., 1976), and Rosaceae (0–0.2 mg/100 g) (Matthaus and Özcan, 2014) have also been reported to contain squalene. Furthermore, presence of squalene in some unconventional oil sources such as apricot kernels (12–43 mg/100 g) (Rudzinska et al., 2017) and borage (22 mg/100 g) (Czaplicki et al., 2011) has also been explored.

In the industrial refining of plant oils, a step called “deodorizing” is usually employed to remove the impurities. At the end of this step, many bioactive compounds such as squalene, tocopherols, phytosterols, and fatty acids accumulate as by-products, commonly called as “deodorizer distillate” (Popa et al., 2015; Sherazi and Mahesar, 2016). Though it is a waste product of the refining process, it comprises up to 80% squalene (Popa et al., 2015), making it a better alternative over the crude plant oil for production of squalene. Olive oil contains about 0.99–12.45 g/kg squalene (Giacometti and Milin, 2001), whereas the deodorizing distillates of olive oil contain 100–300 g/kg squalene (Naziri et al., 2011b). Similarly, soybean, sunflower, canola, and palm fatty acid distillates encompass about **18–55**, 43–45, 30–35, and 2–13 g/kg of squalene, respectively (Dumont and Narine, 2007; Naziri et al., 2011b; Naz et al., 2014). In a similar context, wine lees from wineries can be diverted toward squalene production, thereby improving valorization. A yield of 0.6 ± 0.08 g/kg of squalene can be obtained using dry lees (Naziri et al., 2012). The various plant sources and their reported squalene contents are summarized in Table 1.

**Table 1:** Plant sources of squalene.

**Plant source**	**Concentration (mg/100 g DCW)**	**Reference**
**OILS**		
Amaranth	60,000	Wejnerowska et al., 2013
	46,000	Rosales-García et al., 2017b
	2,000–8,000	Naziri et al., 2011b
	1,040–6,980	He and Corke, 2003
	6,960	Lyon and Becker, 1987
	5,220	Czaplicki et al., 2011
Olive	99–1,245	Giacometti and Milin, 2001
	80–1,200	Lanzón et al., 1994
	250–925	Gutfinger and Letan, 1974
	110–839	Beltrán et al., 2016
	375–652	Nenadis and Tsimidou, 2002
	564	Frega et al., 1992
	170–460	Grigoriadou et al., 2007
	342–450	Manzi et al., 1998
Ginseng seed	514–569	Beveridge et al., 2002
Pumpkin seed	523	Czaplicki et al., 2011
	352.9	Tuberoso et al., 2007
	260–350	Naziri et al., 2011b
Rice bran	320	Rukmini and Raghuram, 1991
	318.9	Pokkanta et al., 2019
Brazil nut	145.8	Derewiaka et al., 2014
Peanuts	132.9	Pokkanta et al., 2019
	127.6	Tuberoso et al., 2007
	27.4	Frega et al., 1992
White sesame seed	60.7	Pokkanta et al., 2019
Black sesame seed	57.2	Pokkanta et al., 2019
Palm	20–50	Goh et al., 1985
	43.3	Lau et al., 2005
Coriander seed	45.1	Pokkanta et al., 2019
Apricot kernel	12.6–43.9	Rudzinska et al., 2017
Hazelnut	9.3–39.2	Bada et al., 2004
	27.9	Frega et al., 1992
	25.7	Derewiaka et al., 2014
Macadamia nut	38.3	Derewiaka et al., 2014
	18.5	Maguire et al., 2004
	7.2–17.1	Wall, 2010
Avocado	34.1–37.0	Gutfinger and Letan, 1974
Corn	33.8	Tuberoso et al., 2007
	30.6	Frega et al., 1992
	10–17	Naziri et al., 2011b
Pecan	29.8	Fernandes et al., 2017
	20.8	Derewiaka et al., 2014
Pistachio	5.5–22.6	Salvo et al., 2017
	8.2	Derewiaka et al., 2014
Borage	22	Czaplicki et al., 2011
Soybean	22	Maguire et al., 2004
	3–20	Naziri et al., 2011b
	18.4	Pokkanta et al., 2019
	12.5–14.3	Gutfinger and Letan, 1974
	9.9	Frega et al., 1992
Sunflower seed	0-19	Naziri et al., 2011b
	17	Tuberoso et al., 2007
Rape seed	43.7	Tuberoso et al., 2007
Grape seed	10.2–16.2	Wen et al., 2016
	14.1	Frega et al., 1992
Cashew	11.6	Derewiaka et al., 2014
Almond	9.6	Fernandes et al., 2017
	1.3	Liu et al., 1976
Cotton-seed	9.10	Gutfinger and Letan, 1974
	2.78	Liu et al., 1976
Flaxseed	1.0–4.2	Tanska et al., 2016
Coconut	1.6	Gutfinger and Letan, 1974
Walnut	0.94	Maguire et al., 2004
	0.09	Liu et al., 1976
Rosaceae seed	0.02–0.29	Matthaus and Özcan, 2014
**DISTILLATES**		
Olive oil	10,000–30,000	Naziri et al., 2011b
	28,000	Bondioli et al., 1993
Soybean oil	5,500	Dumont and Narine, 2007
	1,800–3,500	Naziri et al., 2011b
	1,830	Gunawan et al., 2008
Sunflower oil	4,300–4,500	Naz et al., 2014
Canola oil	3,000–3,500	Naz et al., 2014
Palm fatty acid	200–1,300	Naziri et al., 2011b
	1,030	Posada et al., 2007
Wine lees	6,000	Naziri et al., 2012

*DCW, dry cell weight*.

In spite of holding a good amount of squalene, plants cannot be considered as an ideal source of squalene because some plants are strictly seasonal and the amount of squalene varies greatly geographically. Plants critically require the appropriate temperature and humidity, favorable climatic conditions, soil texture, scheduled irrigation, steady rainfall, fertilizer, and pest management. Aside from the challenges that have been discussed, the process of cultivation is in itself labor intensive and correspondingly the amount of squalene produced from plant sources is not sufficient to fulfill the increasing demand of squalene.

### Squalene From Microorganisms

Microorganisms are amongst the prominent natural sources of squalene. Even though they do not accumulate as much amount as the sharks and plants do, their fast and prodigious growth along with the ease to engineer make them a better alternative for squalene production. The well-studied eukaryotic model organism *Saccharomyces cerevisiae* (yeast) was reported to produce a small amount of squalene, up to 1.6 mg/g dry cell weight (DCW) (Mantzouridou and Tsimidou, 2010; Naziri et al., 2011a). A number of fungi such as *Torulaspora delbrueckii* (0.24 mg/g DCW) (Bhattacharjee et al., 2001), *Aspergillus nidulans* (0.3 mg/g DCW) (Goldberg and Shechter, 1978), *Kluyveromyces lactis* (Drozdíková et al., 2015), and industrial yeast *Saccharomyces uvarum* (14.3 mg/g DCW) have the ability to produce squalene (Blagović et al., 2001).

A newly isolated oleaginous yeast strain *Pseudozyma* sp. JCC 207 is capable of producing squalene up to 340 mg/L (Chang et al., 2008). This yield is considerably better and is suitably high to be used for commercial squalene production. A number of microorganisms such as *Euglena* (Anding et al., 1971), *Candida famata* (Tsujiwaki et al., 1995), *Rhodopseudomonas palustris* (Xu et al., 2016) and archaea *Halobacterium cutirubrum* (1 mg/g DCW) (Goldberg and Shechter, 1978) were also reported to store squalene. Marine bacteria such as *Rubritalea squalenifaciens* sp.nov. (15 mg/g DCW) (Kasai et al., 2007), *R. spongiae* sp.nov. (Yoon et al., 2007), *R. tangerina* sp.nov. (Yoon et al., 2007), and *R. sabuli* sp. nov. (Yoon et al., 2008) belong to the family *Verrucomicrobiaceae* are also known to accumulate squalene.

Apart from that, *Pseudomonas* sp. (Goldberg and Shechter, 1978; Uragami and Koga, 1986), *Methylomonas methanolica* (Goldberg and Shechter, 1978) and *Methylococcus capsulatus* (Goldberg and Shechter, 1978) were found to produce squalene up to 0.76, 1.16, 5 mg/g DCW, respectively. Some microalgae such as *Schizochytrium mangrovei* (0.16 mg/g) (Jiang et al., 2004), *Aurantiochytrium* sp. (0.57–0.72 mg/g DCW) (Li et al., 2009; Chen et al., 2010; Fan et al., 2010) and *Botryococcus braunii* (Banerjee et al., 2002; Uchida et al., 2015) can accumulate considerable amount of squalene.

*Thraustochytrids* are eukaryotic, marine squalene amassing microorganisms that can grow rapidly in harsh conditions when organic carbon is well furnished (Aasen et al., 2016). In a study conducted to screen and characterize squalene accumulating *Aurantiochytrium* sp. strains from the Hong Kong mangrove sites, the strain *Aurantiochytrium* sp. BR-MP4-A1 was found to produce the highest squalene titre (2.46 g/L) after 72 h among all the tested isolates, whereas its squalene content extended up to 0.567 mg/g DCW following 36 h of incubation (Li et al., 2009). Many studies have confirmed that *Aurantiochytrium* sp. could accumulate a considerable amount of squalene (Jiang et al., 2004; Chen et al., 2010; Fan et al., 2010; Kaya et al., 2011; Hoang et al., 2014, 2018; Nakazawa et al., 2014). In a patent (WO/2012/159979) registered for discovering a process for producing squalene from the microalgal species *Thraustochytriales*, claimed that by providing an enriched medium with vitamins B_12_, B_1_ and/or B_6_ at 30°C temperature, squalene titre could be obtained between 20 and 120 mg/g DCW (Pora et al., 2014). Including those discussed, a number of wild-type microorganisms and their reported squalene content are given in Table 2.

**Table 2:** Wild-type microorganisms as a source of squalene.

**Microorganism**	**Squalene**		**Reference(s)**
	**Yield(mg/g DCW)**	**Titre(mg/L)**	
*Saccharomyces cerevisiae*	0.04–1.6	ND	Mantzouridou and Tsimidou, 2010
*Torulaspora delbrueckii*	0.24	ND	Bhattacharjee et al., 2001
*Aurantiochytrium* sp. 18 W-13a	198	1,290	Kaya et al., 2011
*Aurantiochytrium* sp. Yonez 5–1	317.74	1073.66	Nakazawa et al., 2014
*Aurantiochytrium* sp. BR-MP4-A1	0.57	ND	Li et al., 2009
*A. mangrovei*	0.16–120	ND	Jiang et al., 2004; Pora et al., 2014
*Aspergillus nidulans*	0.3	ND	Goldberg and Shechter, 1978
*Halobacterium cutirubrum*	1	ND	Goldberg and Shechter, 1978
*Methylococcus capsulatus*	5.5	ND	Goldberg and Shechter, 1978
*Pseudomonas* sp.	0.10–0.76	ND	Goldberg and Shechter, 1978
*Methylomonas methanolica*	1.16	ND	Goldberg and Shechter, 1978
*Rubritalea squalenifaciens* sp. nov.	15	ND	Kasai et al., 2007
*Kluyveromyce lactis*	0.6 mg/10^9^ cells	ND	Drozdíková et al., 2015
*Pseudozyma* sp. JCC 207	70.32	340.52	Chang et al., 2008

*DCW, dry cell weight; ND, no data*.

The level of squalene can be enhanced and improved in wide range of microorganisms by; (1) using fermentation with additionally optimized conditions (Chen et al., 2010; Fan et al., 2010; Nakazawa et al., 2012), (2) genetic manipulation or introduction of squalene producing gene(s) in native squalene biosynthesis pathways (Bunch and Harris, 1986; Singh et al., 2017, 2018; Valachovič and Hapala, 2017), and (3) addition of an inhibitor (e.g., terbinafine) that blocks the competitive pathway thus allowing squalene accumulation.

## Squalene Biosynthetic Pathway in Microorganisms

Two major isoprenoids pathways have been discovered and used for the production of squalene. It can be either synthesized via the MVA or the MEP pathway, where the latter is also sometimes referred to as the DXP (1-deoxy-D-xylulose-5-phosphate) pathway, depending upon the organism in which the process is taking place. Prokaryotes such as bacteria and cyanobacteria possess the MEP pathway whereas eukaryotes comprising yeast, higher fungi, plants, animals and humans bear the MVA pathway for the synthesis of squalene.

**Figure 2:**
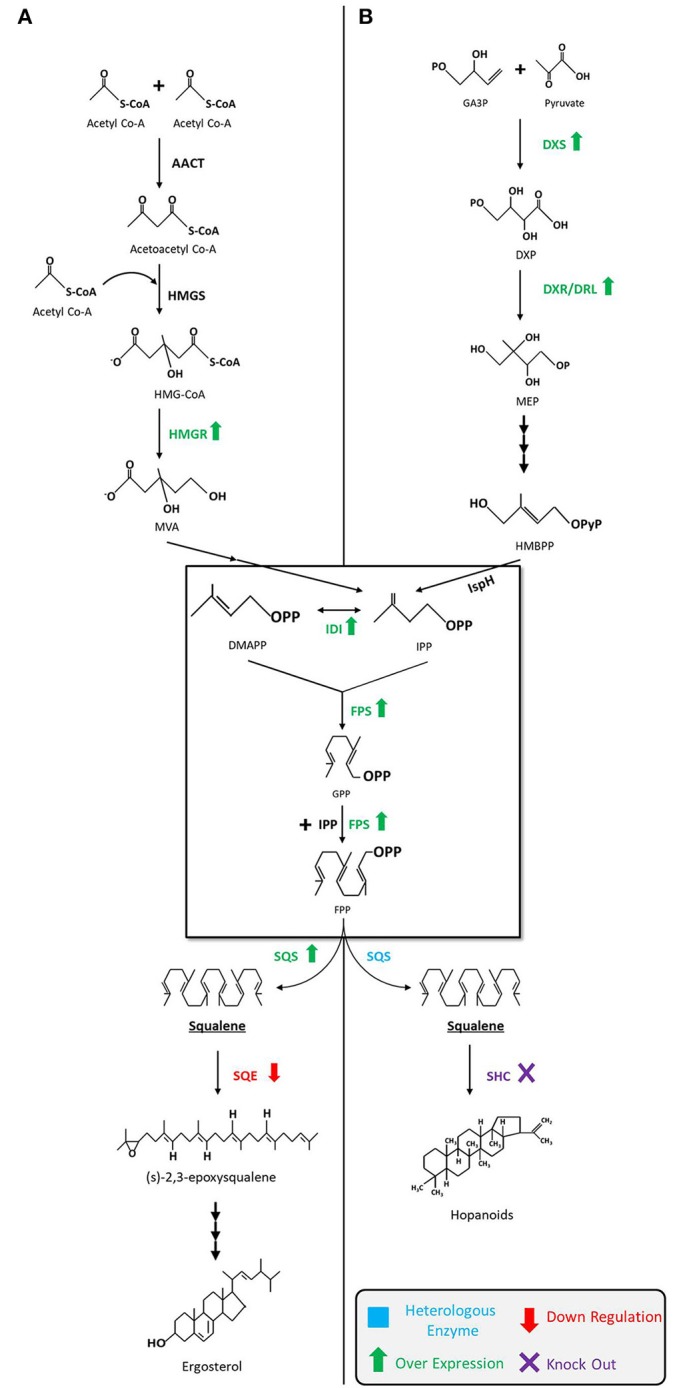
Squalene biosynthetic pathways and production strategies. **(A)** Squalene biosynthesis via MVA pathway in yeast, fungi, and algae. The engineering strategies for enhanced squalene production are as follows: overexpression of *HMGR* (Polakowski et al., 1998; Tokuhiro et al., 2009; Mantzouridou and Tsimidou, 2010; Dai et al., 2012, 2014; Zhuang and Chappell, 2015; Rasool et al., 2016a,b; Kwak et al., 2017; Paramasivan and Mutturi, 2017; Han et al., 2018; Huang et al., 2018; Wei et al., 2018) and *SQS* (Dai et al., 2014; Zhuang and Chappell, 2015; Rasool et al., 2016a,b), downregulation of *SQE* (Garaiová et al., 2014; Hull et al., 2014; Zhuang and Chappell, 2015; Rasool et al., 2016a,b; Han et al., 2018) in yeast; downregulation of *SQE* in algae (Kajikawa et al., 2015). **(B)** Squalene biosynthesis via MEP pathway in bacteria. The engineering strategies for the enhanced squalene production are as follows: overexpression of *DXS* (Ghimire et al., 2009), *IDI* (Ghimire et al., 2009; Katabami et al., 2015) and *FPS* (Katabami et al., 2015), introduction of heterologous squalene producing enzyme(s) (Ghimire et al., 2009; Furubayashi et al., 2014a; Katabami et al., 2015; Pan et al., 2015) in *E. coli*; overexpression of *DXS* and *IDI*, and the introduction of FPS into *Synechococcus elongatus* (Choi et al., 2016); deletion of *SHC* in *Synechocystis* (Englund et al., 2014) and *Rhodopseudomonas palustris* (Xu et al., 2016). MVA, mevalonate; MEP, 2-*C*-methyl-d-erythritol 4-phosphate; AACT, acetoacetyl-CoA thiolase; HMGS, 3-hydroxy-3-methylglutaryl-CoA synthase; HMGR, HMG-CoA reductase; DMAPP, dimethylallyl diphosphate; IDI, isopentenyl diphosphate isomerase; IPP, isopentenyl pyrophosphate; FPS, farnesyl pyrophosphate synthase; GPP, geranyl diphosphate; FPP, farnesyl pyrophosphate; SQS, squalene synthase; SQE, squalene epoxidase; GA3P,glyceraldehyde-3-phosphate; DXP, 1-deoxy-D-xylulose-5-phosphate; DXS, DXP synthase; DXR, DXP reductoisomerase; DLR, DXR-like enzyme; HMBPP, 4-hydroxy-3-methyl-but-2-enylpyrophosphate; IspH,IPP/DMAPP synthase; SHC, squalene hopene cyclase.

As shown in Figure 2, the MVA pathway starts with the condensation of three units of acetyl-CoA to form 3-hydroxy-3-methylglutaryl-CoA (HMG-CoA) via acetoacetyl-CoA, in consecutive reactions catalyzed by the enzymes acetoacetyl-CoA thiolase (AACT) and HMG-CoA synthase (HMGS). Later, HMG-CoA is reduced to MVA in presence of cofactor NADPH by HMG-CoA reductase (HMGR) and then MVA is subsequently phosphorylated twice by enzymes MVA kinase and phospho-MVA kinase to form MVA-5-diphosphate. Following that, decarboxylation of MVA-5-diphosphate takes place in presence of adenosine triphosphate (ATP) to form isopentyl diphosphate (IPP). Next, IPP interconverts into dimethylallyl diphosphate (DMAPP) through a reaction catalyzed by IPP isomerase (IDI). Condensation of both molecules, IPP and DMAPP, by farnesyl diphosphate synthase (FPS) results in geranyl diphosphate (GPP), which is followed by a condensation reaction converting GPP to a 15-carbon isopentyl block unit—FPP (farnesyl pyrophosphate). Eventually, two molecules of FPP are used to synthesize squalene by an NADPH mediated reaction catalyzed by squalene synthase (SQS, encoded by *ERG9*). The pathway then continues onward to synthesize sterols (Ghimire et al., 2016; Mao et al., 2017).

The MEP pathway begins with the condensation of glyceraldehyde-3-phosphate (GA3P) and pyruvate to build DXP that is catalyzed by DXP synthase (DXS). DXP then undergoes reduction to form MEP by DXP reductoisomerase (DXR) or its isozyme DRL (DXR-like). MEP is subsequently transformed into IPP and DMAPP in the subsequent steps by a series of enzymes. The remaining steps from IPP to FPP are identical to the MVA pathway. Unlike the MVA pathway, MEP pathway does not usually produce squalene, but advances in metabolic engineering and synthetic biology techniques have made it possible to design and introduce a heterologous *SQS* gene(s) that can extend the MEP pathway to produce squalene (Ghimire et al., 2009; Katabami et al., 2015).

## Fermentation Optimization For Squalene Production

A wide range of chemicals and bioactive molecules have been identified in the organisms that are produced as an intermediate product in the pathways (Sanchez and Demain, 2008; Keasling, 2010; Chubukov et al., 2016; Panchasara et al., 2018). In order to elevate the industrial scale production of valuable products, fermentation technology uses the adaptability of the natural pathways to produce a desired molecule from the organisms by utilizing cheaper sources of substrates (Saha, 2003). In this way, the input cost can be minimized for large-scale production through the use of particular strains in controlled environments.

Several groups have demonstrated the ability of *S. cerevisiae* to produce squalene, although the amount produced by this strain is considerably low (0.041 mg/g of biomass). Therefore, it is not a suitable candidate for squalene production unless the fermentation conditions are optimized (Bhattacharjee et al., 2001; Fornairon-Bonnefond et al., 2002). In an evaluation of the potential of baker's yeast *S. cerevisiae* and *T. delbrueckii* for fermentative squalene production, a higher amount of squalene (237.25 μg/g DCW) was achieved in *T. delbrueckii* as compared to *S. cerevisiae* (41.16 μg/g DCW). This demonstrates that *T. delbrueckii* could be used for commercial squalene production (Bhattacharjee et al., 2001). Optimizing the effect of oxygen supply, inoculum size, and fermentation time on *S. cerevisiae* BY4741 and EGY48 strains revealed that weaker oxygen supply is more favorable for squalene production. The maximum squalene production was noted to be 2.97 ± 0.12 and 3.13 ± 0.11 mg/L, whilst productivity of **0.10** and **0.16** mg/L/h was gained for *S. cerevisiae* BY4741 and EGY48, respectively (Mantzouridou et al., 2009). Providing anaerobic condition is another proven strategy for enhanced production of squalene in *T. delbrueckii* (Bhattacharjee and Singhal, 2003). By providing a constant flow rate of 0.2 L/min of carbon dioxide (CO_2_), a temperature of 60°C, and pressure of 250–255 bar, squalene yields up to 11.12 μg/g could be obtained using the supercritical fluid extraction (SFE) technique, an extraction method generally benefitting non-polar compounds and low molecular weight compounds (Bhattacharjee and Singhal, 2003). This yield could be further increased up to 430.52 μg/g by performing lyophilization (freeze-drying) prior to SFE (Bhattacharjee and Singhal, 2003). In another study, a mutant strain of *S. cerevisiae* YUG37-*ERG1* yielded about 18.0 ± 4.18 mg/L of squalene when grown in grass juice (*Lolium perenne*) as feedstock with 0.025 μg/ml doxycycline (Hull et al., 2014). Other than *S. cerevisiae* strains, *Kluyveromyces lactis* could also be optimized for increased squalene production (0.6 mg/10^9^ cells) by adding terbinafine (7.5 mg/L) as an inhibitor (Drozdíková et al., 2015).

A potential thraustochytrid *Aurantiochytrium mangrovei* FB3 strain acts as an alternative squalene producer in the fermentation industries through glucose concentration optimization. In an experiment, squalene content was lifted to **2.21 mg/L** with a glucose concentration of 30 g/L. This yield was further elevated by 36 and 40% following treatment of 10 and 100 mg/L terbinafine, respectively, as compared to the control strain (Fan et al., 2010). Through optimization of culture conditions for the strain *Aurantiochytrium* sp. 18W-13a, another group achieved 171 mg/g DCW squalene content (and 0.9 g/L production) by utilizing 2% glucose and 50% seawater concentration in glucose-peptone-yeast medium (GPY medium) incubated at a temperature of 25°C (Nakazawa et al., 2012). Chen et al. (2010) screened various nitrogen sources and found that monosodium glutamate, yeast extract, and tryptone allow improved squalene production. Furthermore, optimal concentrations determined by a central composite experimental design anticipated to recover 6.61, 6.13, and 4.50 mg/L of squalene content and 6.94, 6.22, and 4.40 mg/L of squalene yield, respectively. The results were verified practically by achieving a squalene content of 0.72 mg/g and a squalene yield of 5.90 mg/L for this strain. Likewise, *Schizochytrium mangrovei* was also studied for squalene production using 30 and 150 L bioreactors (Hoang et al., 2014). In the experiment, a squalene content of 33 mg/g DCW was recovered for both cases, while the yields reached 0.99 and 1.01 g/L, respectively (Hoang et al., 2014). Recently, this strain was analyzed for different fermentation conditions, wherein the maximum squalene content (98.07 mg/g of lipid) was attained after 48 h of incubation in a 15 L medium containing 22% glucose (Hoang et al., 2018). High squalene producing novel yeast-like fungus strain *Pseudozyma* SD301 has also been isolated. Highest titre of 2.44 g/L squalene was achieved using fed-batch fermentation by providing glucose and yeast extracts to a ratio of 3, maintaining the system at pH 6 and 25°C, with supplementation of 15 g/L of sea salt (Song et al., 2015).

Agricultural by-products are great economical and sustainable sources for fermentation and for the extraction of bio-compounds. Very recently, Fagundes et al. (2018) demonstrated an excellent finding by growing the cyanobacteria *Phormidium autumnale* in a bubble-column bioreactor with procured industrial slaughterhouse wastewater, accomplishing squalene amount totaling up to 0.18 mg/g DCW. Whilst the production they achieved was not otherwise sufficient industrially, they demonstrated a positive finding in this study by achieving squalene from waste and rubbish. The fermentation conditions and their optimization parameters are given in Table 3. Alteration or overproduction accomplished by genetic engineering of the microbes is discussed in the upcoming section.

**Table 3:** Fermentation optimization for squalene production.

**Microorganism**	**Conditions**	**Fermentation** ** volume/mode**	**Squalene**		**Reference**
			**Yield** **(mg/g DCW)**	**Titre** **(g/L)**	
*S. cerevisiae*	Nutrients (GPY medium), 30°C temp., pH 5.5. Optimized: inoculum size (5%), incubation period (48 h), anaerobic conditions	100 mL shake flask	1.38	ND	Bhattacharjee et al., 2001
*T. delbrueckii*	Nutrients (GPY medium), 30°C temp., pH 5.5. Optimized: inoculum size (5%), incubation period (24 h), anaerobic conditions	100 mL shake flask	1.89	ND	Bhattacharjee et al., 2001
*S. cerevisiae* EGY48	Nutrients (glucose, yeast extract, and soy peptone). Optimized: terbinafine (0.44 mM) plus methyl jasmonate (0.04 mM) for squalene content, terbinafine (0.30 mM) for squalene yield	100 mL shake flask	10.02	0.020	Naziri et al., 2011a
*S. cerevisiae* BY4741	Nutrients (glucose, soy peptone, yeast, and malt extracts), 30°C temp., pH 5.5, 200 rpm. Optimized: oxygen supply (low), inoculum size (5%), incubation time (28.5 h)	100 mL shake flask	ND	2.96^*^10^−3^	Mantzouridou et al., 2009
	Nutrients (glucose, soy peptone, yeast, and malt extracts), 30°C temp., pH 5.5, 200 rpm. Optimized: oxygen supply (low), inoculum size (8%), incubation time (45 h)	100 mL shake flask	ND	3.12^*^10^−3^	
*T. delbrueckii*	Nutrients (glucose, yeast extract, peptone), pH 5.5, anaerobic, 30°C temp. Optimized: temp.60°C, pressure 250–255 bar and 0.2 L/min CO_2_flowSFE technique	2.5 L shake flask	0.01	ND	Bhattacharjee and Singhal, 2003
	Nutrients (glucose, yeast extract, peptone), pH 5.5, anaerobic, 30°C temp. Optimized: lyophilization prior to SFE under the above mentioned conditions	2.5 L shake flask	0.43	ND	
*K. lactis*	Nutrients (YPL medium). Optimized: terbinafine (7.5 mg/L)	ND	0.6 mg/10^9^ cells	ND	Drozdíková et al., 2015
*A. mangrovei* FB3	Nutrients (GPY medium), 25°C temp., inoculum size 5%. Optimized: glucose (30 g/L)	100 mL shake flask	0.37	2.21^*^10^−3^	Fan et al., 2010
*Aurantiochytrium* sp. strain 18W-13a	Nutrients (GPY medium), 25°C temp., 100 rpm. Optimized: Incubation time (96 h)	ND	198	1.29	Kaya et al., 2011
*Aurantiochytrium* sp. strain 18W-13a	Nutrients (GPY medium), 130 rpm. Optimized: temp. 25°C, seawater (25–50%), glucose (2–6%)	200 mL shake flask	171	0.9	Nakazawa et al., 2012
*Aurantiochytrium* sp. BR-MP4-A1	Nutrients (glucose, yeast extract,salts), temp. 25°C, pH 6, inoculum size 5%, 200 rpm, dark. Optimized: N-source (monosodium glutamate (6.61–6.94 g/L), yeast extract (6.13–6.22 g/L), tryptone (4.40–4.50 g/L))	50 mL shake flask	0.72	5.90^*^10^−3^	Chen et al., 2010
*Schizochytrium mangrovei*PQ6	Nutrients: (M12 medium: glucose, yeast, artificial sea water), inoculum size 2–3%, temp. 28°C, pH 6.5–7.5	15 L	33.00 ± 0.02	0.99	Hoang et al., 2014
	Nutrients: (M12 medium: glucose, yeast, artificial sea water), inoculum size 2–3%, temp. 28°C, pH 6.5–7.5	100 L	33.04 ± 0.03	1.01	
*S. mangrovei*PQ6	Nutrients (glucose, yeast extract, urea, salts). Optimized: fermentation mode (fed-batch), incubation time (48 h)	15 L fed-batch fermentation	98.07 mg/g of lipid	ND	Hoang et al., 2018
*Pseudozyma* SD301	Nutrients (GPY medium). Optimized: temp. 25°C, pH 6, carbon (glucose), nitrogen (yeast extract), C/N ratio (3), sea salt (15 g/L)	50 mL shake flask for optimization, 3.5L for fed-batch fermentation	ND	2.44	Song et al., 2015
*Phormidium autumnale*	Industrial slaughterhouse wastewater, C/N ratio 30, temperature 26°C, pH 7.6, keptdark	Bubble column bioreactor	0.18	ND	Fagundes et al., 2018

*DCW, dry cell weight; ND, no data; temp, temperature; GPY, glucose peptone yeast; C/N, carbon/nitrogen; rpm, revolutions per minute; YPL, yeast peptone lactose; SFE, supercritical fluid extraction*.

## Engineering of Microorganisms for Squalene Production

As microorganisms have rapid growth and insightful genetic background, they are easy to manipulate and use as a squalene microbial cell factories. In addition, quantum leap in molecular biology, synthetic biology, metabolic engineering and genome engineering have opened up new avenues for the production of numerous valuable chemicals, biofuels, and important metabolites from renewable sources (Rabinovitch-Deere et al., 2013; Singh et al., 2014, 2016; Chubukov et al., 2016; Gohil et al., 2017). By recognizing the minute aspects of the metabolic pathways and their flux, positive modifications around squalene producing pathways can be modulated to increase the synthesis of desired compounds.

### Engineering *Saccharomyces cerevisiae* for Squalene Production

The accessibility and availability of wide range gene manipulating toolboxes allow easy manipulation of *S. cerevisiae* for industrial applications. In 1994, the first squalene accumulating *S. cerevisiae* was engineered through the disruption of a gene required for the conversion of squalene to ergosterol by homologous recombination in ergosterol biosynthesis pathway (EBP) of *S. cerevisiae*. As ergosterol is an integral constituent of the fungal plasma membrane, the generated mutants required this compound for faster growth. Accordingly, by feeding ergosterol into the system, squalene production could be increased up to 5 mg/g DCW under aerobic condition (Kamimura et al., 1994). In yeast, the *ERG1* gene encodes a key enzyme of interest called squalene epoxidase, which is responsible for the conversion of squalene to squalene epoxide in the EBP. This *ERG1* gene is a potent target of genetic manipulation toward increasing squalene production in yeast (Garaiová et al., 2014; Hull et al., 2014). Even point mutations in the *ERG1* gene enable to achieve a higher amount of squalene yield (up to 1 mg/10^9^ cells) without any growth retardation (Garaiová et al., 2014). Thus, the resultant mutants could be hyper-sensitized to terbinafine, a squalene epoxidase inhibitor. Supporting this theory, Hull et al. (2014) developed a mutant strain *S. cerevisiae* YUG37-ERG1, which features a doxycycline-repressible tet0_7_-CYC1 promoter that regulates *ERG1* gene expression, making the organism capable of producing up to 7.85 mg/g squalene following addition of 50 μg/mL doxycycline.

Another strategy for increasing the metabolic flux toward squalene in *S. cerevisiae* is to overexpress the genes which encode the rate-limiting enzymes (e.g., HMGR, ERG9) that are encountered in the MVA biosynthesis pathway. Yeast has two genes, *HMG1* and *HMG2*, which encode HMGR. Overexpression of *HMG1* gene has shown to overproduce squalene up to 191.9 mg/L (Tokuhiro et al., 2009). Similarly, Mantzouridou and Tsimidou (2010) investigated the effect of genetic perturbations in *HMG2* and *ERG6* (encodes sterol 24-C-methyltransferase that converts zymosterol to fecosterol) genes on squalene accumulation in *S. cerevisiae* EGY48. They constructed an AM63 mutant strain that harbors an additional copy of the *HMG2* gene with a K6R stabilizing mutation in Hmg2p, an HMGR isoenzyme. Furthermore, they also created an AM64 strain by deletion of *ERG6* gene from the AM63 strain. As a consequence, the AM63 mutant could produce squalene up to 18.3 mg/g DCW after 12 h of incubation that was 20-fold higher than the control strain. Incidentally, the AM64 mutant could not accumulate squalene further, as it diverts surplus squalene into C27 sterols. Hmg1p is another important NADPH dependent membrane-bound isomer of HMGR that contributes for more than 83% of enzyme activity in the wild-type strain (Basson et al., 1986; Paramasivan and Mutturi, 2017). By integrating double copies of truncated *HMG1* (*tHMG1*) genes, squalene accumulation could be increased by 16.8-fold in contrast to a single-copy of a gene in *S. cerevisiae* (Paramasivan and Mutturi, 2017). The tHMG1p enzyme requires NADPH cofactor, which is also an electron carrier in yeast, and accordingly the production could be further enhanced by 27.5-fold (28.4 ± 1.08 mg/L) through the co-expression of both *tHMG1* and *POS5* (encodes an enzyme for NADPH regeneration) genes (Paramasivan and Mutturi, 2017). By implementing overexpression of the *ERG9* gene along with insertion mutations in the *ERG1* gene, 85 mg/L squalene has been produced in *S. cerevisiae* BY4741 (Zhuang and Chappell, 2015). Additionally, this has been further improved to **270** mg/L by expressing the truncated *HMGR* (*tHMGR*) gene (Zhuang and Chappell, 2015). Similarly, Polakowski et al. (1998) overexpressed the HMGR by constructing and expressing the *tHMG1* gene under the control of a constitutive *ADH1* promoter. The resulting strain exhibited 2-fold increase in HMGR activity and 40-fold greater accumulation of squalene as compared to the parental strain *S. cerevisiae* AH22. Dai et al. (2014) performed similar experiments where they integrated *tHMG1* with an addition of the *LYS2* gene in *S. cerevisiae* BY4742-TRP, obtaining a significant amount of squalene (150.9 mg/L). Toward further increasing squalene accumulation, the β-amyrin synthase (*bAS*) gene from *Glycyrrhiza glabra* was introduced with *ERG9* and *ERG1* genes. This emanated strain yielded 183.4 mg/L squalene. In addition, Kwak et al. (2017) co-expressed the *tHMG1* and *ERG10* (encodes Acetyl-CoA C-acetyltransferase) genes in *S. cerevisiae* SR7 (a xylose-utilizing engineered strain) and obtained up to 532 mg/L squalene within 54 h of incubation period in xylose fed-batch fermentation.

Overexpression of the entire squalene biosynthetic pathway in *S. cerevisiae* has also been demonstrated. Genes *tHMG1, IDI1* (encodes isopentenyl-diphosphate-isomerase), *ERG20* (FPS), and *ERG9* were overexpressed under the control of *HHF2p, IRA1p, PET9p*, and *RHO1p* promoters, respectively, leading to an increased squalene production by 35.02-fold (119.08 mg/L) when treated with terbinafine (Rasool et al., 2016a). Eventually, the complete biosynthetic pathway for squalene was overexpressed and that obtained a yield reaching as high as **304.49** mg/L (Rasool et al., 2016a). It was noted during the investigation that overexpression of the squalene biosynthetic pathway downregulates both ethanol production as well as post-squalene biosynthetic pathways (Rasool et al., 2016a).

An unexpected squalene accumulation (78 mg/L) was observed when Dai et al. (2012) inserted a gene to produce miltiradiene, a precursor of a well-known Chinese traditional medicine called tanshinone. Their attempt to overexpress tHMGR and upc2.1 (a mutated regulatory factor that induces sterol biosynthetic gene) in *S. cerevisiae* BY4742 to yield miltiradiene in upcoming steps surprisingly led to over accumulation of squalene as an intermediate product. Very recently, Han et al. (2018) overexpressed *tHMG1* and *ispA* (bacterial FPP synthase) genes in *S. cerevisiae* Y2805 strain and obtained squalene concentration up to 400 ± 45 mg/L. They further increased squalene yield up to 756 ± 36 mg/L by the inclusion of terbinafine for partial inhibition of SQE. Subsequently, they employed large-scale fed-batch fermentation and produced a yield of 2,011 ± 75 and 1,026 ± 37 mg/L squalene with and without supplementation of terbinafine, respectively.

Strength of the promoter also plays a crucial role for balancing, tuning and optimizing the expression of a gene toward enhancing the metabolite concentrations. In this perspective, Rasool et al. (2016b) overexpressed the genes present in the squalene biosynthetic pathway with a newly characterized and optimized library of 13 new constitutive promoters in *S. cerevisiae* INVSc1. The resultant engineered strain FOH-0 produced up to 100 mg/L squalene that was 29.41-fold higher than the control strain. They further improved squalene production in the FOH-2 strain by upregulating the entire squalene biosynthetic pathway, achieving 304.16 mg/L squalene in presence of terbinafine.

Wei et al. (2018) illustrated an approach for increasing the lipid content in yeast in order to increase squalene production. Accordingly, they overexpressed *tHMG1* and *DGA1*, the latter being the gene that encodes diacylglycerol acyltransferase (a triacylglycerols biosynthesis enzyme), and deleted the *PXA2* (a subunit of peroxisomal ABC transport complex involved in the transportation of long-chain fatty acids into peroxisomes) and *POX1* (Fatty acyl-coA oxidase) genes to reduce lipid β-oxidation. The resultant strain boosted squalene accumulation by 250-fold as compared to the control strain *S. cerevisiae* D452-2. However, it was noted that deletion of *PXA2* and *POX1* genes failed to give additional squalene enhancement, and did not help to accumulate the product of interest. In order to achieve a high titre, they performed fed-batch fermentation and obtained 445.6 mg/L squalene from nitrogen restricted minimal medium.

All of the above studies show promising data for the improvement of squalene production toward the industrial applications. These engineered strains can be optimally grown in cheaper carbon and nitrogen from renewable and sustainable sources for the industrial scale production of squalene at more competitive prices than many alternatives.

### Engineering *Escherichia coli* for Squalene Production

*E. coli* is a well-known model organism for the study of genetics, physiology, biochemistry, molecular biology, and many more. A reason why *E. coli* can be a lucrative source of squalene production is due to its inability to produce endogenous triterpenoids, owing to which the strains can stock up large amounts of biosynthesized squalene as they avert its conversion to undesired compounds. To facilitate this, single or multiple genes that pertain to squalene biosynthesis can be integrated into the MEP pathway.

**Figure 3:**
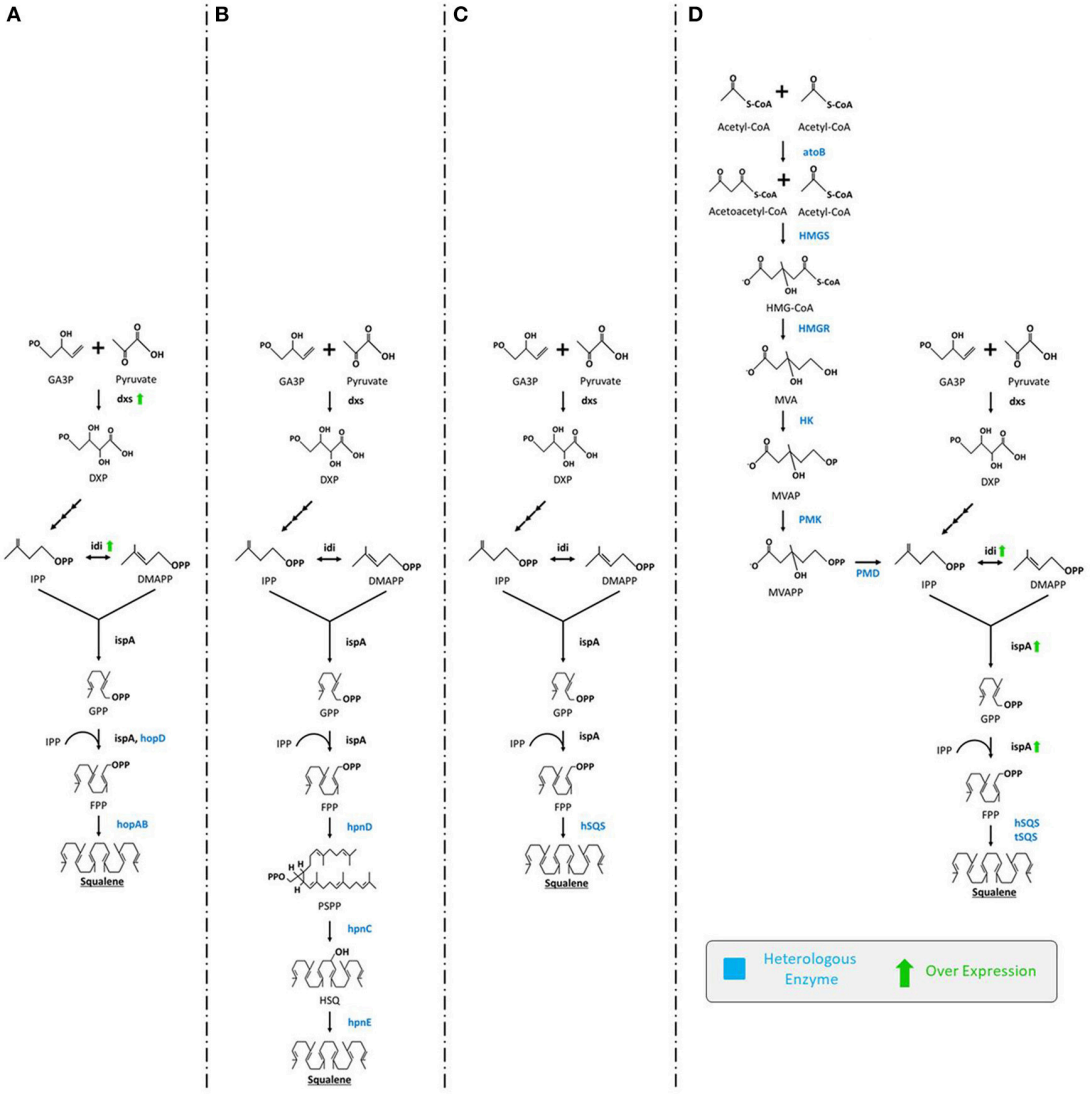
Various engineering strategies employed for enhanced production of squalene in *E. coli*. **(A)** Ghimire et al. (2009) expressed *hopD* and *hopAB* from *Streptomyces peucetius* followed by overexpression of *dxs* and *idi*, **(B)** Pan et al. (2015) expressed *hpnC, hpnD*, and *hpnE* from *Zymomonas mobilis* or *Rhodopseudomonas palustris* **(C)** Furubayashi et al. (2014a) expressed *hSQS*, **(D)** Katabami et al. (2015) expressed *hSQS* or *tSQS* from *Thermosynechococcus elongates, atoB, HMGS, HMGR, HK, PMK, PMD* from *S. cerevisiae* followed by overexpression of *idi* and *ispA*. GA3P, glyceraldehyde-3-phosphate; DXP, 1-deoxy-D-xylulose-5-phosphate; dxs, DXP synthase; IPP, isopentenyl pyrophosphate; idi, isopentenyl diphosphate isomerase; DMAPP, dimethylallyl diphosphate; ispA, farnesyl pyrophosphate synthase; GPP, geranyl diphosphate; FPP, isopentenyl pyrophosphate; hopD, farnesyl diphosphate synthase; hopAB, squalene/phytoene synthases; hpnC, hydroxyl squalene synthase; hpnD, presqualene diphosphate synthase; hpnE, hydroxysqualene dehydroxylase; hSQS, human squalene synthase; tSQS, *Thermosynechococcus* SQS; atoB, acetyl-CoA acetyltransferase; HMGS, 3-hydroxy-3-methylglutaryl-CoA synthase; HMGR, HMG-CoA reductase; HK, mevalonate kinase; PMK, phosphomevalonate kinase; PMD, mevalonate diphosphate decarboxylase.

An ample number of reports describe engineering of *E. coli* for the production of squalene through the extension of MEP biosynthetic pathway by inserting squalene synthase gene (*SQS*). In the first systematic study, three hopanoid genes including *hopA, hopB* (encodes squalene/phytoene synthases), and *hopD* (encodes farnesyl diphosphate synthase) from *Streptomyces peucetius* ATCC 27952 were successfully inserted and expressed in *E. coli*. The engineered *E. coli* was able to produce squalene up to 4.1 mg/L (Ghimire et al., 2009). There are number of rate limiting genes (e.g., *idi, dxs, dxr*) in the MEP pathway that could be overexpressed in order to improve squalene production. Inserting additional copies of *dxs* and *idi* genes using plasmids for overexpression of rate limiting enzymes improved squalene production up to 11.8 mg/L (Figure 3A; Ghimire et al., 2009). In a similar study, Pan et al. (2015) introduced a three-step MEP extended squalene biosynthetic pathway in *E. coli* that included *hpnC, hpnD*, and *hpnE* genes from *Zymomonas mobilis* and *Rhodopseudomonas palustris*. The pathway begins with enzyme hpnD catalyzing the conversion of two molecules of FPP to presqualene diphosphate (PSPP), followed by the PSPP being transformed into hydroxy-squalene (*HSQ*) and subsequently to squalene by enzymes hpnC and hpnE, respectively. This engineered strain could produce significant amounts of squalene (Figure 3B).

Insertion of a single squalene synthase gene (encoding *SQS*) can also catalyze the conversion of two molecules of FPP (the end-product of the MEP pathway) into squalene (Figure 3C) (Furubayashi et al., 2014a; Katabami et al., 2015). It has been noted that SQS is NADPH dependent, a rate-limiting factor in cellular systems (Furubayashi et al., 2014a). This strategy also clearly requires codon-optimization. In a related study, the efficiency of two dissimilar codon-optimized *SQS*, namely human (*hSQS*) and *Thermosynechococcus* (*tSQS*) squalene synthases were analyzed in *E. coli* XL1-Blue strain. Between them, the *hSQS* expressed in the bacterial cells were found to produce squalene (4.2 mg/L). For further enhancement, the whole MVA pathway from *S. cerevisiae* was also introduced in *E. coli* for the very first time. Briefly, codon-optimized *tHMGR, ERG13* (encodes hydroxymethylglutaryl-CoA synthase), *ERG12* (mevalonate kinase), *ERG8* (phosphomevalonate kinase), and *MVD1* (mevalonate diphosphate decarboxylase) from *S. cerevisiae* were introduced along with the overexpression of *idi* and *ispA* (farnesyl diphosphate synthase). From this strain squalene content obtained was elevated up to 230 and 150 mg/L using *hSQS* and *tSQS*, respectively (Figure 3D; Katabami et al., 2015). The study of the truncation effect on *SQS* revealed that the C-terminus retains partial cellular activity, sufficient to produce squalene (Katabami et al., 2015).

For indirect monitoring of squalene production, a CrtN based biosensor has been developed by transforming the *hSQS* gene along with the *CrtN* gene (dehydrosqualene desaturase) from *Staphylococcus aureus*. As a consequence, yellow colored pigmentation of the cells could be obtained because of the conversion of squalene into yellow carotenoid pigment (Furubayashi et al., 2014a). These results can also be used to assist the screening of cellular activity of different *SQS* genes (Furubayashi et al., 2014a).

Protein engineering is currently a powerful tool for improving enzyme activity, substrate specificity, and evolutionary fitness through the evolution of the enzymes of interest. Directed evolution of *SQS* genes was performed to determine the activity and evolutionary adaptability of the gene that could allow improved production of squalene (Furubayashi et al., 2014b). This study demonstrated that the direct evolution of different *SQS* genes from *S. cerevisiae*, humans, and *Thermosynechococcus elongatus* BP-1 could exhibit numerous beneficial mutations that could expand the activity of SQS (Furubayashi et al., 2014b).

As aforementioned, in *S. cerevisiae*, the immediate product after squalene is squalene epoxide which is the precursor of the paramountly important compound ergosterol that forms the basic unit of a fungal plasma membrane. Blocking or disrupting the pathway following squalene synthesis, owing to the accumulation of squalene leads to the death of the fungus (Jandrositz et al., 1991). Unlike *S. cerevisiae, E. coli* does not utilize squalene for further terpenoids conversion, making it a preferred choice of strain for many investigators. Additionally, the mindful targeting and downregulation of complementary pathways could also prove to be a suitable strategy to mitigate the resultant metabolic burden (Huang et al., 2018).

### Engineering *Cyanobacteria, Algae*, and Other Microorganisms for Squalene Production

Genome sequencing of cyanobacteria *Synechocystis* sp. PCC 6803 (Kaneko and Tabata, 1997) revealed that it contains a complete set of genes that are required to produce hopanoids through the MEP pathway. In this pathway, squalene plays an important role as a precursor for hopanoids. Disabling *shc* gene (encodes squalene hopene cyclase which converts squalene to hopene) in *Synechocystis* could lead to accumulation of squalene (0.67 mg/L/OD_750_) in the generated mutant strain without any detrimental effect upon cell growth (Englund et al., 2014). In another study, Choi et al. (2016) engineered cyanobacteria *Synechococcus elongatus* PCC 7942 to convert it into biosolar-cell factories for production of squalene and amorpha-4,11-diene from CO_2_, resulting in significant production of squalene (4.98 mg/L/OD_730_). They overexpressed three important rate-limiting genes (*dxs, idi*, and *dxr*) of the MEP pathway along with the *E. coli ispA* gene in different combinations, finding that the mutant strain SeSC33S (*dxs, idi, ispA* overexpressed) was capable of producing a high amount of squalene (4.98 ± 0.90 mg/L/OD_730_). A study looking at the fusion protein CpcB1-SQS found that its expression produced up to 7.16 ± 0.05 mg/L/OD_730_ squalene (Choi et al., 2017). This output was further enhanced up to 11.98 ± 0.49 mg/L/OD_730_ through increasing the gene dosage (encoding CpcB1-SQS) using a strong endogenous *cpcB1* promoter (Choi et al., 2017). Moreover, a scalable yield was achieved (7.08 ± 0.5 mg/L/OD_730_, ~79.2 mg/g DCW) by using a 6 L CO_2_-fed bag-type photo-bioreactor with optimized conditions of 5% CO_2_ under 100 μmol photons/m^2^/s (Choi et al., 2017).

Other than cyanobacteria, Xu et al. (2016) used the purple non-sulfur bacteria *Rhodopseudomonas palustris* TIE-1 for squalene production. They hypothesized and subsequently demonstrated that blocking *shc* gene (converts the squalene to the hopanoid) in the hopanoid pathway can aid accumulation of squalene in this strain. Investigators could enhance squalene production by 27-fold (3.8 mg/g DCW) as compared to the control strain. They could further improve the production of squalene up to 9.9 mg/g DCW by increasing carbon-flux toward FPP (the precursor of squalene) via co-expression of genes *crtE* and *hpnD*. Interestingly, when they fused the proteins CrtE and HpnD by a linker molecule (GGGGS)_3_, squalene content increased up to 12.6 mg/g DCW. Finally, they overexpressed the rate-limiting enzyme DXP synthase (encoded by *dxs*) of the MEP pathway and produced 15.8 mg/g DCW that drove a 112-fold increase as compared to the wild-type strain.

Increasing the squalene precursor pool size is also an attractive strategy. As an alternative strategy, oleaginous yeast *Yarrowia lipolytica* was engineered in a way that it would supply the requisite amount of precursors to itself for improving squalene yield (Huang et al., 2018). The metabolite pool size of acetyl-CoA (the start point of the MVA pathway) was increased by overexpressing the codon-optimized heterogeneous *acs* gene (encoding acetyl-CoA synthase) from *Salmonella enterica*, and coupling this with the endogenous *ylACL1* gene (ATP citrate lyase) resulted in a 50% acetyl-CoA enhancement. When this desired acetyl-CoA rich MVA pathway was boosted by overexpressing *ylHMG1* gene, 3.3 mg/g DCW squalene was produced. The level of squalene was enhanced up to 7 and 10 mg/g following supplementation of 20 mM sodium acetate and 10 mM citrate, respectively (Huang et al., 2018).

In addition to this, Kajikawa et al. (2015) exploited the transgenic *Chlamydomonas reinhardtii* to investigate the squalene accumulation capacity of the microalgae by characterizing microalgal squalene synthase (CrSQS) and squalene epoxidase (CrSQE). Overexpression of the *CrSQS* illustrated that CrSQS increases the rate of conversion of FPP to squalene. However, it failed to accumulate more squalene content. Furthermore, they noted that knocking down of *CrSQE*, resulted in squalene accumulation being enhanced by up to 1.1 mg/g DCW. Thus, it was suggested that the partial suppression of *CrSQE* is an effective strategy for squalene accumulation in *C. reinhardtii*. The various engineering strategies along with their achieved squalene outputs are given in Table 4.

**Table 4:** Squalene production in engineered microorganisms.

**Microorganisms**	**Strategy**	**Squalene**		**Reference**
		**Content (mg/g DCW)**	**Yield (mg/L)**	
*S. cerevisiae* SHY3	Disruption of a gene involved in the conversion of squalene to ergosterol by homologous recombination	5	ND	Kamimura et al., 1994
*S. cerevisiae* BY4741	Point mutations in *ERG1*, the gene responsible for conversion of squalene to squalene epoxide, thereby promoting hypersensitivity to terbinafine	1 mg/10^9^ cells	ND	Garaiová et al., 2014
*S. cerevisiae* YUG37	Regulation of *ERG1* expression by promoter *tet0*_7_ *-CYC1*	7.85 ± 0.02	ND	Hull et al., 2014
*S. cerevisiae* YPH499	Overexpression of *HMG1* (encodes HMGR)	ND	191.9	Tokuhiro et al., 2009
*S. cerevisiae* EGY48	Overexpression of *HMG2* with a K6R stabilizing mutation in Hmg2p, an HMGR isoenzyme	18.3	ND	Mantzouridou and Tsimidou, 2010
*S. cerevisiae* BY4741	Overexpression of *tHMG1* and *POS5* with mitochondrial presequence	58.6 ± 1.43	28.4 ± 1.08	Paramasivan and Mutturi, 2017
	Overexpression of *tHMG1* and *POS5* without mitochondrial presequence	33.0 ± 2.96	46.0 ± 4.08	
*S. cerevisiae* BY4741	Overexpression of *ERG9* and *POS5* without mitochondrial presequence	ND	85	Zhuang and Chappell, 2015
	Overexpression of *ERG9*and *tHMGR*, insertion mutation in *ERG1*	ND	270	
*S. cerevisiae* AH22	Overexpression of *tHMG1* under constitutive promoter	ND	ND	Polakowski et al., 1998
*S. cerevisiae* BY4742-TRP	Overexpression of *tHMG1, LYS2*	ND	150.9	Dai et al., 2014
	Overexpression of *tHMG1, LYS2, ERG9, ERG1*, expression of *bAS* (b-amyrin synthase) from *Glycyrrhiza glabra*	ND	183.4	
*S. cerevisiae* SR7	Co-expression of*tHMG1* and *ERG10* gene in xylose-rich medium	ND	532	Kwak et al., 2017
*S. cerevisiae* Y2805	Overexpression of *tHMG1*, expression of *ispA*	ND	400 ± 45	Han et al., 2018
	Overexpression of *tHMG1*, expression of *ispA*, fed-batch fermentation	ND	1026 ± 37	
	Overexpression of *tHMG1*, expression of *ispA*, fed-batch fermentation with supplementation of terbinafine	ND	2011 ± 75	
*S. cerevisiae* BY4742	Overexpression of *tHMGR* and *upc2.1* (a mutated regulatory factor that induces sterol biosynthetic gene)	ND	78	Dai et al., 2012
*S. cerevisiae* INVSc1	Overexpression of *tHMG1, IDI1* (isopentenyl diphosphate-isomerase), *ERG20* (farnesyl diphosphate synthase), and *ERG9*	ND	34	Rasool et al., 2016a
	Overexpression of *tHMG1, IDI1, ERG20*, and *ERG9*, supplementation of terbinafine	ND	119.08	
	Overexpression of *tHMG1, IDI1, ERG20, ERG9, ERG10* (encoding acetyl-CoA C-acetyltransferase)*, ERG13* (HMG-CoA synthase)*, ERG12* (mevalonate kinase)*, ERG8* (phosphomevalonate kinase), and *MVD1* (diphosphomevalonate decarboxylase)	ND	304.49	
*S. cerevisiae* INVSc1	Overexpression of squalene biosynthetic pathway using a library of 13 new constitutive promoters	ND	100	Rasool et al., 2016b
	Overexpression of squalene biosynthetic pathway using a library of 13 new constitutive promoters, supplementation of terbinafine	ND	304.16	
*S. cerevisiae* D452-2	Overexpression of *tHMG1* and *DGA1*, fed-batch fermentation in nitrogen restricted minimal media	ND	445.6	Wei et al., 2018
*E. coli* BL21(DE3)	Expression of *hopA* and *hopB* (squalene/phytoene synthases) together with *hopD* (farnesyl diphosphate synthase) from *Streptomyces peucetius*	ND	4.1	Ghimire et al., 2009
	Overexpression of *dxs* and *idi* (rate limiting enzymes), expression of *hopA* and *hopB* together with *hopD* from *Streptomyces peucetius*	ND	11.8	
*E. coli*	Expression of *hpnC, hpnD*, and *hpnE* from *Zymomonas mobilis*	ND	ND	Pan et al., 2015
	Expression of *hpnC, hpnD*, and *hpnE* from *Rhodopseudomonas palustris*	ND	ND	
*E. coli* XL1-Blue	Expression of human *SQS* (*hSQS*)	ND	4.2	Katabami et al., 2015
	Co-expression of *hSQS*, chimeric mevalonate pathway containing *tHMGR, ERG13* (hydroxymethylglutaryl-CoA synthase), *ERG12* (mevalonate kinase), *ERG8* (phosphomevalonate kinase) and *MVD1* (mevalonate diphosphate decarboxylase) from *S. cerevisiae*, overexpression of *atoB* (acetyl-CoA acetyltransferase), *idi* (isoprenyl diphosphate isomerise) and *ispA* (farnesyl diphosphate synthase)	54	230	
	Co-expression of *Thermosynechococcus elongatus SQS* (*tSQS*), chimeric mevalonate pathway containing *tHMGR, ERG13, ERG12, ERG8*, and *MVD1* from *S. cerevisiae*, overexpression of *atoB, idi*, and *ispA*	55	150	
*E. coli* XL1-Blue	Expression of *hSQS*	ND	2.7 mg/L	Furubayashi et al., 2014a
*Synechocystis* sp. PCC 6803	Disabling *shc* (squalene hopene cyclase)	ND	0.67 /OD_750_	Englund et al., 2014
*Synechococcuselongatus* PCC 7942	Overexpression of *dxs* and *idi*, expression of *ispA* from *E. coli*	ND	4.98 ± 0.90 /OD_730_	Choi et al., 2016
*S. elongatus* PCC 7942	Expression of CpcB1-SQS protein	ND	7.16 ± 0.05/OD_730_	Choi et al., 2017
	Increased gene dosage of CpcB1-SQS by strong endogenous *cpcB1* promoter	ND	11.98 ± 0.49 /OD_730_	
*Rhodopseudomonas palustris* TIE-1	Disabling *shc*	3.8	ND	Xu et al., 2016
	Disabling *shc* gene, co-expression of *crtE* and *hpnD*	12.6	ND	
	Disabling *shc* gene, co-expression of *crtE* and *hpnD*, overexpression of *dxs*	15.8	ND	
*Yarrowia lipolytica*	Overexpression of *acs* (from *Salmonella enterica*), ylACL1 (encodes acetyl-CoA synthase), and *ylHMG1*	3.3	ND	Huang et al., 2018
	Overexpression of *acs* (from *Salmonella enterica*), ylACL1 (encodes acetyl-CoA synthase), and *ylHMG1*, addition of 20 mM sodium acetate	7	ND	
	Overexpression of *acs* (from *Salmonella enterica*), ylACL1 (encodes acetyl-CoA synthase), and *ylHMG1*, addition of 10 mM citrate	10	ND	
*Chlamydomonas reinhardtii* C-9	Overexpression of *CrSQS*, knocked down *CrSQE*.	0.9-1.1	ND	Kajikawa et al., 2015

*HMGR, HMG-CoA reductase; tHMG1, truncated HMG1; tHMGR, truncated Hydroxymethylglutaryl-CoA reductase*.

The future holds great possibilities to attempt engineering microbes, as there remain many unexplored strains to date that may show improved options for the synthesis of the desired product. However, this may bring along several unintended risk factors such as chances of interbreeding, impact on the ecosystem, enhanced selection pressure on organisms and/or horizontal transfer of recombinant genes to other microbes during conjugation (Prakash et al., 2011). A probable way to avoid such ascertainable risks is by following the International biosafety regulatory frameworks (Prakash et al., 2011).

## Potential Applications of Squalene

**Figure 4:**
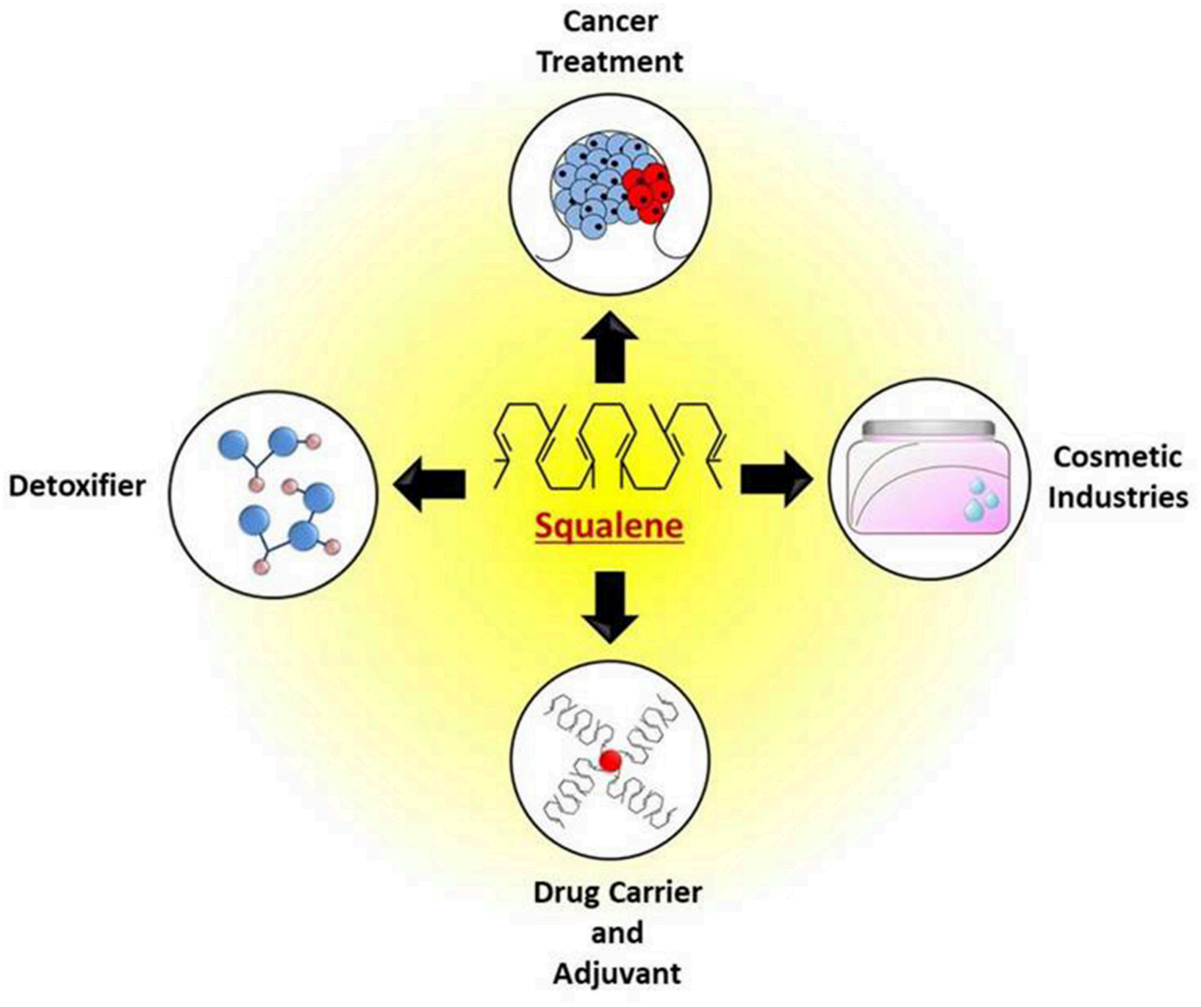
Potential applications of squalene for treatment of cancer, as detoxifier, use in cosmetics, drug and vaccine adjuvants.

Squalene has been used for a wide range of applications and has huge impacts on our daily life. It has the ability to serve as a powerful antioxidant, anti-cancer agent, vehicle to transport drugs and vaccines, and its role in skincare and personal care products undoubtedly underscores the beneficial effects of squalene. The Japanese have been designating squalene as “Tokubetsu no Miyage” meaning “a precious gift,” acknowledging it as a source of strength, power, and vitality. Locals of the Izu peninsula of Japan have been using squalene to treat numerous diseases and conditions, owing to which they designated squalene as “Samedawa,” meaning “cure-all” (Popa et al., 2015). The inherent applications of squalene in nutraceutical, pharmaceutical, a drug carrier, detoxifier etc. are shown in Figure 4.

### Squalene as an Anticancer Agent

Cancer is one of the most dreadful diseases that mankind faces, and it generally manifests as uncontrolled growth of cells that can metastasize into other parts of the body. A wide range of studies have shown that the regular consumption of squalene may inhibit the proliferation of tumor cells, especially those of the breast, pancreas, colon, and melanoma, the activation of which depends on the prenylation of proteins (Newmark, 1997; Cirmena et al., 2018).

Many oncogenes are involved in cancer initiation and progression, amongst which the *Ras* oncogene plays a leading role in initiating cancer by transforming healthy cells into malignant one (Fernández-Medarde and Santos, 2011). Under the physiological conditions, the *Ras* gene functions as a switch that regulates the cell growth, but in tumor cells, the hyperactivated *Ras* gene promotes the autonomous growth of cells. Ras proteins are required to undergo isoprenylation during their activation and anchorage to the cell membrane (Fernández-Medarde and Santos, 2011). During the cell division when the cells transit from the G1 phase to S phase, some vital isoprenylated proteins translocate to the cell membrane as well as to the nuclear envelope. The isoprenylation of these proteins is facilitated by GPP and FPP, which in turn are regulated by squalene. Dysregulated isoprenylation of certain proteins affects the Ras signal transduction process in actively proliferating and differentiating cells. Therefore, it proves to be calamitous, especially for the *Ras* hyperactivated tumor cells, as well as the normal cells (Rao et al., 1998; Smith, 2000). A dietary intervention of squalene inhibits isoprenoid production by setting up a negative feedback in response to the presence of exogenous squalene (Ronco and De Stéfani, 2013). The inhibition takes place because down-regulation of HMGR decreases the synthesis of FPP, thus suspending the MVA pathway and thereby inhibiting the isoprenylation of Ras proteins. By arresting the cells at the G1 phase of the cell cycle, squalene prevents the proliferation of cancer cells.

In several reports, cancer has been linked with the feeding style of the population in question. A diet rich in squalene, antioxidants, and fiber has been associated with a decline in the mortality rate amongst the populations that consume a high amount of olive oil. Particularly, the Mediterranean diet that includes a fair share of squalene and phenolic compounds from the consumption of fish and olive oil lowers the incidence of degenerative diseases (Newmark, 1997; Owen et al., 2004). According to Smith (2000), squalene restricts carcinogenesis by three identified mechanisms. Firstly, it inhibits the action of HMGR and prevents the conversion of HMG-CoA into MVA. Secondly, it tightly regulates the metabolism of xenobiotic compounds by modulating the biosynthesis of the enzymes that are involved in the process. Thirdly, squalene can scavenge the free radicals and singlet reactive oxygen that are responsible for causing a mutagenic lesion in the DNA, ultimately leading to cancer.

Early reports have suggested that a combination therapy of anti-cancer drugs emulsified with squalene have shown to intensify the efficacy of drugs both directly and indirectly (Yarkoni and Rapp, 1979). Moreover, the increase in the beneficial cytotoxic effect of potent drugs such as adriamycin, 5-fluorouracil, bleomycin, and cisplatin on administration with squalene has already been reported (Nakagawa et al., 1985). The response of certain drugs, when co-administered with squalene, can greatly differ between breast cancer cells and normal mammary epithelial cells. Warleta et al. (2010) studied the effect of squalene on cell proliferation, apoptosis, oxidative stress, and cellular damage resulting from reactive oxygen species (ROS) in human breast cells. From their findings, they deduced that squalene reduced the levels of intracellular ROS and oxidative damage, generated *in vitro* by H_2_O_2_, in normal epithelial cells but not in breast tumor cells.

Similarly, it was demonstrated that squalene has the ability to selectively protect the normal cells against the cytotoxic effect of the anti-cancer drugs, but it does not extend the same protective mechanism to tumor cells (Das et al., 2003). In the subsequent study, Das et al. (2008) presented the experimental evidence of squalene's role in protecting healthy bone marrow cells while having no obvious effect on the neuroblastoma cells. A related study in rats that were each fed 0.4 mL/day of squalene signified the protective role of this triterpene on cyclophosphamide-induced toxicity in the kidney, liver, and heart of the rats (Senthilkumar et al., 2006a,b).

Rapid DNA synthesis is a hallmark of cancer. To design anti-cancer therapeutics that target the process, pro-drug strategies dealing with quick and efficient transportation of nucleotide analogs have been employed (Couvreur et al., 2006). Originally designed to be a powerful anti-viral tool, these nucleotide analogs are significantly different from their nucleotide counterparts and can inhibit DNA synthesis in rapidly dividing cells (Couvreur et al., 2006). However, the permeability of these nucleotide analogs is restricted due to their hydrophobicity and unfavorable pharmacodynamics. Squalenylation is the process of coating anticancer or antiviral drugs with squalene to increase the permeability and cytotoxic activity, and it helps in overcoming the described limitations by greatly increasing the intracellular accessibility of these compounds (Couvreur et al., 2006). An example of this is the enhanced membrane permeability and interaction of the gemcitabin-squalene complex as compared to free gemcitabin (Castelli et al., 2007; Pili et al., 2010; Peramo et al., 2018).

### Squalene as an Antioxidant

Squalene has long been recognized as a potential antioxidant candidate. It has the capacity to entrap thermodynamically active singlet oxygen atoms that are generated from auto-hydrolytic reactions and resultant oxidative products (Pham et al., 2015). The chemical structure of squalene shares close resemblance with lycopene (β-carotene), the carotenoid pigment responsible for red color of many fruits and vegetables, as well as some endogenous antioxidants including glutathione, superoxide dismutase, ubiquinone (coenzyme Q10), and vitamins A, E, and K1, all of which are notable antioxidants (Huang et al., 2009; Ronco and De Stéfani, 2013). The six unconjugated double bonds of squalene make it a highly sustainable oxygen-scavenging agent that is more stable against oxidation arising from peroxides.

Prolonged exposure to sunlight or ultra-violet (UV) radiation by any means gives rise to highly reactive free radicals in the system, leading to biomembrane destruction, premature skin aging, and other associated diseases (Kohno et al., 1995). Moreover, the human skin is not as hairy as other primates and therefore is prone to undergo rapid peroxidation of endogenous lipids through exposure. In the skin, squalene may act as a powerful quencher of singlet oxygen, thereby preventing the lipid peroxidation (Kohno et al., 1995; Kelly, 1999). Data from the photoluminescence studies of squalene using mass spectrometry revealed that it is the double bonds of the squalene that binds with oxygen and prevent photooxidation by UV rays (Mudiyanselage et al., 2003). Squalene has the prodigious ability to take up a greater amount of free oxygen as compared to any other lipid present in the human skin (Kohno et al., 1995). It has the ability to take up about one-fourth of its weight of oxygen, allowing squalene to prevent the development of wrinkles, acne, and comedones (Huang et al., 2009).

A number of studies support the fact that three major phenolic constituents of olive oil (simple phenols like hydroxytyrosol and tyrosol, secoiridoids, and lignans) along with squalene, vitamin E, and monounsaturated fatty acid (oleic acid) offer protection against many cancers and coronary heart diseases (Owen et al., 2000a,b). Intake of >13.5 g/day of squalene is proven to reduce the facial wrinkling, enhance the type I procollagen expression, and decrease the facial erythema along with substantially reducing the risk of UV-induced damage to the DNA (Murakoshi et al., 1992; Cho et al., 2009).

The metabolism of a number of anticancer drugs often releases highly toxic free radicals. Cyclophosphamide, also known as cytophosphane, and chloroacetaldehyde, are metabolized in the kidney. The nascent oxygen released from their metabolism generates oxidative stress in the kidney leading to its early damage (Rehman et al., 2012). Adriamycin (Doxorubicin) is known to form superoxide anions that are pernicious to cardiac tissues (Rahman et al., 2007). Apart from these, the anticancer drugs that bear platinum can affect bone marrow over long-term use. Squalene may help to neutralize the adverse effect of these drugs by absorbing the free radicals and pacifying the oxidative stress.

### Squalene in the Skin and Personnel Care

Considered amongst the best-known emollients, squalene is one of the common components of the human sebum and the compatibility it shares with lipids present in the skin explains its extensive use in the cosmetic industries. Squalene, together with its hydrogenated analog squalane, has the ability to occlude moisture in the dermal layers, thus counteracting the appearance of fine lines and dry patches. It penetrates into deeper layers of skin and provides elasticity, restores suppleness, and drastically improves the flexibility. Moreover, though technically a lipid, squalene leaves little oily residues and is safe to use on sensitive and acne-prone skin (Sethi et al., 2016).

The ability of squalene to serve as a quencher of singlet oxygen and sustain lipid photo-peroxidation explains its role in the formulations of anti-aging creams and sunscreens. Using harsh skin cleansers, not only eliminate the dirt but also rob moisture from the skin. Squalene has been found to reverse the elevated transepidermal water loss (TEWL) along with riboflavin penetration in sodium lauryl sulfate (SLS)-treated rats and human skin (Okuda et al., 2002). Besides being an excellent hydrating agent, topical applications of squalene-based products are used to treat skin disorders including acne, psoriasis, xerosis-related skin lesions, seborrheic dermatitis, and atopic dermatitis (Wołosik et al., 2013; Hon et al., 2018). An *in vivo* study on the delivery of psoralen, an anti-psoriatic medicine, suggests that entrapment of the drug inside a nanostructured lipid carrier based on squalene and precirol facilitates better skin permeability and controlled release of the drug (Fang et al., 2008).

Rissmann et al. (2008) developed a semi-synthetic lipid formulation that could mimic the composition, organization, and thermotropic behavior of vernix caseosa (VC) lipids, presuming their role in acting as a protective barrier, as it does for the human fetus. A set of mixtures were generated by mixing varying lipid fractions isolated from lanolin with squalene, triglycerides, cholesterol, ceramides, and fatty acids which resulted in improved barrier recovery in barrier-deficient skin (i.e., for psoriasis). Other than the applications of squalene in emollients and moisturizers, squalene can aid the quick dispersion of the dyes from lipsticks, resulting in a glossy finish and potentially prolonging the long-lasting fragrances by fixing the perfumes. Squalene when applied over the damaged or washed hair and skin can readily form an emulsion with the oils or other lipophilic substances. It may prevent them from turning rancid, thereby helping to restore the natural oil (Wołosik et al., 2013). Given the properties of squalene as a natural emollient and moisturizer, the scope for squalene to find more application in the cosmetic sector is widespread and immense.

### Squalene as a Drug and Vaccine Carrier

Squalene has been used for the formulation of drug delivery and as a vaccine adjuvant candidate. Owing to its lipophilicity and excellent surface tension, squalene has been extensively used for preparing the stable oil-in-water emulsions for the purpose of delivering active hydrophobic compounds. The slow release of these active compounds enhances the biodistribution and aids improved uptake by the cells (Naziri et al., 2011b). Moreover, reports suggest that the ease with which a lipid carrier interacts with endogenous body fluid and tissues readily decreases the possibility of adverse effects (Nicolaos et al., 2003; Huang et al., 2009). Squalene emulsions possess a high degree of transfection activity and are known to delineate very low systemic toxicity following intravenous administration *in vivo* (Kim et al., 2003). Additionally, squalene in comparison to other lipids can form fairly stable emulsions that facilitate the delivery of a lipophilic drug when loaded into the discontinuous oil phase (Chung et al., 2001).

Squalene prepared into emulsion either as the lone ingredient or in combination with other components can readily improve the functioning of a drug and may also help to elicit a favorable immune response against the exogenous antigen (Kedl and Kedl, 2015). A lecithin-squalene emulsion in conjugation with Tween 80 (polysorbate 80) has been found to be effective in stimulating the antibody production, while squalene emulsion alone was found to assist the efficacy for an influenza vaccine (10 mg squalene/dose) (WHO, 2006; Kim and Karadeniz, 2012). In a recent study, a group of researchers developed a squalene-containing solid lipid nanoparticle formulation. This formulation was designed keeping yeast vaccines in mind and comes with added advantages of steam sterilization and the fact that it can be freeze-dried and stored in a powdered form coupled with the vaccine. The designed complex could successfully produce immune stimulating effects that were comparable to Freund's adjuvant and the commercially available squalene nanoemulsion AddaVax™ (Stelzner et al., 2018).

*In vivo* studies on rat models have helped to generate substantial evidence supporting the use of squalene emulsions with phosphatidylethanolamine or Pluronic® F68 for improved parenteral drug delivery of morphine and its prodrugs. The slow release of drug prolongs the analgesic effect in the preclinical model. The antinociceptive activity analyzed through the cold ethanol tail-flick test revealed that as compared to the aqueous formulations, the lipid-based emulsions exhibit an extended analgesic response (Wang et al., 2008).

Several controversies have revolved around the use of squalene as an adjuvant for the anthrax vaccine (Asa et al., 2000). A downside of using squalene was debated following its relation to treatments of Gulf War Syndrome (GWS) that included a pool of symptoms such as fatigue, headache, diarrhea, rashes and allergies, memory loss and neurological abnormalities, arthralgias, myalgias, and lymphadenopathy (Gronseth, 2005; Lippi et al., 2010). The unexpected finding of the anti-squalene antibodies in patients with GWS spiked speculation about its safety and its adverse effects (Asa et al., 2002). However, these are now regarded as uncorroborated findings given the fact that humans naturally possess anti-squalene antibodies and therefore the presence of the mentioned antibodies may not contribute to, or indicate the development of GWS (Matyas et al., 2004). This conjecture was further strengthened with the approval of a squalene-based MF59 emulsion adjuvant that neither raised the anti-squalene antibody levels nor modified the pre-existing titres (Del Giudice et al., 2006).

MF59 is potent oil in water-based emulsion designed by Novartis®, with about 4.3% squalene present in the dispersed phase, and surfactants including Span85, Tween 80, and citrate constituting the continuous phase. The emulsion has worked well for several vaccines including malaria (Patra et al., 2015), herpes virus (Hensel et al., 2017), hepatitis B and C viruses (Del Giudice et al., 2006), H1N1 flu (Lippi et al., 2010), and HIV (Del Giudice et al., 2006). Although the exact biological mechanism is not yet clear, it is predicted that MF59 elicits an immune response by recruiting the phagocytic cells to the site of delivery, promoting maturation of monocytes into dendritic cells, and facilitating their transport to nearby lymph nodes to trigger the adaptive immune response (Seubert et al., 2008; Sánchez-Quesada et al., 2018). In the near future, more research needs to be done for achieving the full potential of squalene as a carrier in drug and vaccine for controlling of many serious diseases.

### Squalene as a Detoxifier

Human body tends to accumulate a large amount of xenobiotics (i.e., man-made complex chemicals not found in nature) that are hazardous and can have detrimental effects on health. These compounds interfere with the normal physiological functioning by either acting as potential pro-carcinogens or by mimicking the receptor structure of sex hormones (Omiecinski et al., 2011; Mackowiak and Wang, 2016). Some are inherently non-toxic but turn hazardous following biotransformation by the hepatic cytochrome P450 enzymes (Ioannides et al., 2004). These chemical compounds are usually lipophilic in nature and they are compelled to be drawn toward fat. Squalene being nonpolar in nature has a high affinity for such unionized compounds and therefore facilitates the removal of xenobiotic compounds from the body (Kelly, 1999).

In an attempt to clear the xenobiotic compounds, liver breaks down the noxious products into smaller ones. Squalene stored in the fat cells can act as a detoxifying agent by clearing these compounds. The accumulation of xenobiotics in the fat cells frees the squalene conserved within the cells, which gets released into the circulation. This, in turn, accelerates clearance by stimulating bile secretion (Günes, 2013). A diet supplemented with 8% of squalene has been found to improve the faecal elimination of organochlorine xenobiotics including hexachlorobiphenyl (6-CB) and hexachlorobenzene (HCB) (Richter and Schäfer, 1982; Richter et al., 1982). A few other toxins such as dibenzofurans, 12-O-Tetradecanoylphorbol-13-acetate (TPA) and 4-(Methylnitrosamino)-1-(3-pyridyl)-1-butanone (NNK) (Murakoshi et al., 1992) are also known to be detoxified by squalene. Animal studies reveal that squalene also enhances the removal of xenobiotics such as theophylline, strychnine, and phenobarbital (Kamimura et al., 1992; Kelly, 1999). Hence, it can be presumed that the detoxification power of squalene is a function of its ability to purify and clean the biological systems.

## Concluding Remarks and Perspectives

Squalene shows an impressive range of human applications to date, ranging from a protective anti-cancer (Kim and Karadeniz, 2012; Günes, 2013) and anti-oxidant (Amarowicz, 2009; Günes, 2013) compound to one that assists cosmetics, pharmaceutical drugs, and vaccines and drug delivery (Del Giudice et al., 2006; Pasquale et al., 2015), augmenting their potency and selectivity. Acknowledging its wide range of uses, squalene has been called “Tokubetsu no Miyage” or “a precious gift” in Japanese, and it is clear why. Due to its biological properties and uses in numerous commercial formulations, its synthesis and recovery show great global demand that is reflected in its high market value (Global Market Insights, 2016; Rosales-Garcia et al., 2017a). This continues to grow and is projected to advance further with time, especially if novel applications of squalene are discovered. Clearly with the potency of squalene in so many varied formulations, it would likely find use in new formulations that would demonstrate similar benefits, and the future of squalene is full of novel applications awaiting discovery.

While living up to its Japanese name as a biologically precious gift, the biggest immediate hurdle in expanding the growth of squalene supplies and markets are its primary sources, and specifically their link with the decimation of shark populations (Ciriminna et al., 2014; Tsoi et al., 2016). Given the ethical and social concerns around this topic, the likelihood that sharks should, or even could, remain the primary source of squalene is doubtful (Dulvy et al., 2008). On weighing the benefit of squalene against bioconservation and protection of sharks, we feel that this should compose an overly great challenge, as modern science has advanced to a position where mankind can subvert biological systems genetically, and thereby improve and exploit existing strains that do not suffer bioconservation or ethical use problems. Squalene is produced from only two pathways biologically, the MEP and MVA pathways (Spanova and Daum, 2011; Popa et al., 2015; Rani et al., 2018). Within the pathways and their contexts, biologists and biochemists have modified, truncated, duplicated, deleted, and improved numerous targets, showing positive increase in squalene accumulation. Likewise, naturally producing species (excluding sharks) have been examined, ranging between microbes, plants, and fungi, demonstrating a wide choice of alternative target organisms for the synthesis and accumulation of this compound.

Currently, the amounts of squalene recovered from any alternative source are pale in comparison to sharks, but the potential for further improvement in these species and their fermentation remains interesting, and perhaps more importantly, plausible. Interesting findings in this context include the oleaginous yeast *Pseudozyma* sp. JCC 207, which provides considerable yields of squalene (Chang et al., 2008) that approach commercially viable levels. Likewise, the fungus *T. delbrueckii* has also been shown to synthesize commercially relevant amounts of squalene from fermentation and the SFE technique (Bhattacharjee et al., 2001; Bhattacharjee and Singhal, 2003). Alternatively, the discovery of cheaper and more renewable sources can reduce the barrier to entry for alternative squalene sources, such as cyanobacteria *P. autumnale*, which could use literal waste and rubbish as a source to generate low amounts of squalene (Fagundes et al., 2018).

In the majority of cases examined, the use of fermentation increased the yields and titres of squalene, and this could be improved through various optimization practices and the use of specific inhibitors, such as terbinafine. With the recent and rapid growth in synthetic biology, the ability to modify the MEP/MVA pathway as well as other competing pathways around squalene could be a means to attain improved squalene biosynthesis. The primary concern will drive the microorganisms and the alternative species of choice toward higher yields and titres, something that will likely come through the incremental steps of improvement that will ultimately, and hopefully, reach commercially viable levels. The discovery of new species that may have more malleable biosynthesis pathways or higher native squalene content could also be a priority, as in any case the major and primary source of this incredible compound is not sustainable, both ecologically as well as commercially. Having reviewed the incredible range of applications of squalene, and the exciting work and accomplishments to increase its yield from sustainable biological sources, the description of squalene as an ultimate “precious gift” seems more and more achievable.

## Data Availability

All datasets generated for this study are included in the manuscript and/or the supplementary files.

## Author Contributions

NG, GB, KK, DB, and VS have designed and written the manuscript. VS has supervised and finalized the final version of the manuscript.

### Conflict of Interest Statement

The authors declare that the research was conducted in the absence of any commercial or financial relationships that could be construed as a potential conflict of interest.

## Abbreviations

AACT: Acetoacetyl-CoA thiolase
DMAPP: Dimethylallyl diphosphate
DXP: 1-deoxy-D-xylulose-5-phosphate
FPP: Farnesyl pyrophosphate
FPS: Farnesyl pyrophosphate synthase
GA3P: Glyceraldehyde-3-phosphate
GPP: Geranyl diphosphate
HMBPP: 4-hydroxy-3-methyl-but-2-enylpyrophosphate
HMGR: HMG-CoA reductase
HMGS: 3-hydroxy-3-methylglutaryl-CoA synthase
IDI: Isopentenyl diphosphate isomerase
IPP: Isopentenyl pyrophosphate
MEP: 2-*C*-methyl-d-erythritol 4-phosphate
MVA: Mevalonate
SHC: Squalene hopene cyclase
SQE: Squalene epoxidase
SQS: Squalene synthase
DCW: Dry cell weight.
