# Corrigendum: Engineering Strategies in Microorganisms for the Enhanced Production of Squalene: Advances, Challenges and Opportunities

**DOI:** 10.3389/fbioe.2019.00114

**Published:** 2019-05-28

**Authors:** Nisarg Gohil, Gargi Bhattacharjee, Khushal Khambhati, Darren Braddick, Vijai Singh

**Affiliations:** ^1^School of Biological Sciences and Biotechnology, Institute of Advanced Research, Koba Institutional Area, Gandhinagar, India; ^2^Department of R&D, Cementic S. A. S., Genopole, Paris, France

**Keywords:** squalene, metabolic engineering, fermentation, biosynthesis, production, synthetic biology, anti-oxidant, anti-aging

In the original article, there were mistakes in Tables 1, 3, and 4.

**Table 1:** Plant sources of squalene.

**Plant source**	**Concentration** ** (mg/100 g DCW)**	**Reference**
**OILS**		
Amaranth	60,000	Wejnerowska et al., 2013
	46,000	Rosales-García et al., 2017b
	2,000–8,000	Naziri et al., 2011b
	1,040–6,980	He and Corke, 2003
	6,960	Lyon and Becker, 1987
	5,220	Czaplicki et al., 2011
Olive	99–1,245	Giacometti and Milin, 2001
	80–1,200	Lanzón et al., 1994
	250–925	Gutfinger and Letan, 1974
	110–839	Beltrán et al., 2016
	375–652	Nenadis and Tsimidou, 2002
	564	Frega et al., 1992
	170–460	Grigoriadou et al., 2007
	342–450	Manzi et al., 1998
Ginseng seed	514–569	Beveridge et al., 2002
Pumpkin seed	523	Czaplicki et al., 2011
	352.9	Tuberoso et al., 2007
	260–350	Naziri et al., 2011b
Rice bran	320	Rukmini and Raghuram, 1991
	318.9	Pokkanta et al., 2019
Brazil nut	145.8	Derewiaka et al., 2014
Peanuts	132.9	Pokkanta et al., 2019
	127.6	Tuberoso et al., 2007
	27.4	Frega et al., 1992
White sesame seed	60.7	Pokkanta et al., 2019
Black sesame seed	57.2	Pokkanta et al., 2019
Palm	20–50	Goh et al., 1985
	43.3	Lau et al., 2005
Coriander seed	45.1	Pokkanta et al., 2019
Apricot kernel	12.6–43.9	Rudzinska et al., 2017
Hazelnut	9.3–39.2	Bada et al., 2004
	27.9	Frega et al., 1992
	25.7	Derewiaka et al., 2014
Macadamia nut	38.3	Derewiaka et al., 2014
	18.5	Maguire et al., 2004
	7.2–17.1	Wall, 2010
Avocado	34.1–37.0	Gutfinger and Letan, 1974
Corn	33.8	Tuberoso et al., 2007
	30.6	Frega et al., 1992
	10–17	Naziri et al., 2011b
Pecan	29.8	Fernandes et al., 2017
	20.8	Derewiaka et al., 2014
Pistachio	5.5–22.6	Salvo et al., 2017
	8.2	Derewiaka et al., 2014
Borage	22	Czaplicki et al., 2011
Soybean	22	Maguire et al., 2004
	3–20	Naziri et al., 2011b
	18.4	Pokkanta et al., 2019
	12.5–14.3	Gutfinger and Letan, 1974
	9.9	Frega et al., 1992
Sunflower seed	0-19	Naziri et al., 2011b
	17	Tuberoso et al., 2007
Rape seed	43.7	Tuberoso et al., 2007
Grape seed	10.2–16.2	Wen et al., 2016
	14.1	Frega et al., 1992
Cashew	11.6	Derewiaka et al., 2014
Almond	9.6	Fernandes et al., 2017
	1.3	Liu et al., 1976
Cotton-seed	9.10	Gutfinger and Letan, 1974
	2.78	Liu et al., 1976
Flaxseed	1.0–4.2	Tanska et al., 2016
Coconut	1.6	Gutfinger and Letan, 1974
Walnut	0.94	Maguire et al., 2004
	0.09	Liu et al., 1976
Rosaceae seed	0.02–0.29	Matthaus and Özcan, 2014
**DISTILLATES**		
Olive oil	10,000–30,000	Naziri et al., 2011b
	28,000	Bondioli et al., 1993
Soybean oil	5,500	Dumont and Narine, 2007
	1,800–3,500	Naziri et al., 2011b
	1,830	Gunawan et al., 2008
Sunflower oil	4,300–4,500	Naz et al., 2014
Canola oil	3,000–3,500	Naz et al., 2014
Palm fatty acid	200–1,300	Naziri et al., 2011b
	1,030	Posada et al., 2007
Wine lees	6,000	Naziri et al., 2012

DCW, dry cell weight.

**Table 3:** Fermentation optimization for squalene production.

**Microorganism**	**Conditions**	**Fermentation** ** volume/mode**	**Squalene**		**Reference**
			**Yield** **(mg/g DCW)**	**Titre** **(g/L)**	
*S. cerevisiae*	Nutrients (GPY medium), 30°C temp., pH 5.5. Optimized: inoculum size (5%), incubation period (48 h), anaerobic conditions	100 mL shake flask	1.38	ND	Bhattacharjee et al., 2001
*T. delbrueckii*	Nutrients (GPY medium), 30°C temp., pH 5.5. Optimized: inoculum size (5%), incubation period (24 h), anaerobic conditions	100 mL shake flask	1.89	ND	Bhattacharjee et al., 2001
*S. cerevisiae* EGY48	Nutrients (glucose, yeast extract, and soy peptone). Optimized: terbinafine (0.44 mM) plus methyl jasmonate (0.04 mM) for squalene content, terbinafine (0.30 mM) for squalene yield	100 mL shake flask	10.02	0.020	Naziri et al., 2011a
*S. cerevisiae* BY4741	Nutrients (glucose, soy peptone, yeast, and malt extracts), 30°C temp., pH 5.5, 200 rpm. Optimized: oxygen supply (low), inoculum size (5%), incubation time (28.5 h)	100 mL shake flask	ND	2.96^*^10^−3^	Mantzouridou et al., 2009
	Nutrients (glucose, soy peptone, yeast, and malt extracts), 30°C temp., pH 5.5, 200 rpm. Optimized: oxygen supply (low), inoculum size (8%), incubation time (45 h)	100 mL shake flask	ND	3.12^*^10^−3^	
*T. delbrueckii*	Nutrients (glucose, yeast extract, peptone), pH 5.5, anaerobic, 30°C temp. Optimized: temp.60°C, pressure 250–255 bar and 0.2 L/min CO_2_flowSFE technique	2.5 L shake flask	0.01	ND	Bhattacharjee and Singhal, 2003
	Nutrients (glucose, yeast extract, peptone), pH 5.5, anaerobic, 30°C temp. Optimized: lyophilization prior to SFE under the above mentioned conditions	2.5 L shake flask	0.43	ND	
*K. lactis*	Nutrients (YPL medium). Optimized: terbinafine (7.5 mg/L)	ND	0.6 mg/10^9^ cells	ND	Drozdíková et al., 2015
*A. mangrovei* FB3	Nutrients (GPY medium), 25°C temp., inoculum size 5%. Optimized: glucose (30 g/L)	100 mL shake flask	0.37	2.21^*^10^−3^	Fan et al., 2010
*Aurantiochytrium* sp. strain 18W-13a	Nutrients (GPY medium), 25°C temp., 100 rpm. Optimized: Incubation time (96 h)	ND	198	1.29	Kaya et al., 2011
*Aurantiochytrium* sp. strain 18W-13a	Nutrients (GPY medium), 130 rpm. Optimized: temp. 25°C, seawater (25–50%), glucose (2–6%)	200 mL shake flask	171	0.9	Nakazawa et al., 2012
*Aurantiochytrium* sp. BR-MP4-A1	Nutrients (glucose, yeast extract,salts), temp. 25°C, pH 6, inoculum size 5%, 200 rpm, dark. Optimized: N-source (monosodium glutamate (6.61–6.94 g/L), yeast extract (6.13–6.22 g/L), tryptone (4.40–4.50 g/L))	50 mL shake flask	0.72	5.90^*^10^−3^	Chen et al., 2010
*Schizochytrium mangrovei*PQ6	Nutrients: (M12 medium: glucose, yeast, artificial sea water), inoculum size 2–3%, temp. 28°C, pH 6.5–7.5	15 L	33.00 ± 0.02	0.99	Hoang et al., 2014
	Nutrients: (M12 medium: glucose, yeast, artificial sea water), inoculum size 2–3%, temp. 28°C, pH 6.5–7.5	100 L	33.04 ± 0.03	1.01	
*S. mangrovei*PQ6	Nutrients (glucose, yeast extract, urea, salts). Optimized: fermentation mode (fed-batch), incubation time (48 h)	15 L fed-batch fermentation	98.07 mg/g of lipid	ND	Hoang et al., 2018
*Pseudozyma* SD301	Nutrients (GPY medium). Optimized: temp. 25°C, pH 6, carbon (glucose), nitrogen (yeast extract), C/N ratio (3), sea salt (15 g/L)	50 mL shake flask for optimization, 3.5L for fed-batch fermentation	ND	2.44	Song et al., 2015
*Phormidium autumnale*	Industrial slaughterhouse wastewater, C/N ratio 30, temperature 26°C, pH 7.6, keptdark	Bubble column bioreactor	0.18	ND	Fagundes et al., 2018

*DCW, dry cell weight; ND, no data; temp, temperature; GPY, glucose peptone yeast; C/N, carbon/nitrogen; rpm, revolutions per minute; YPL, yeast peptone lactose; SFE, supercritical fluid extraction*.

**Table 4:** Squalene production in engineered microorganisms.

**Microorganisms**	**Strategy**	**Squalene**		**Reference**
		**Content (mg/g DCW)**	**Yield (mg/L)**	
*S. cerevisiae* SHY3	Disruption of a gene involved in the conversion of squalene to ergosterol by homologous recombination	5	ND	Kamimura et al., 1994
*S. cerevisiae* BY4741	Point mutations in *ERG1*, the gene responsible for conversion of squalene to squalene epoxide, thereby promoting hypersensitivity to terbinafine	1 mg/10^9^ cells	ND	Garaiová et al., 2014
*S. cerevisiae* YUG37	Regulation of *ERG1* expression by promoter *tet0*_7_ *-CYC1*	7.85 ± 0.02	ND	Hull et al., 2014
*S. cerevisiae* YPH499	Overexpression of *HMG1* (encodes HMGR)	ND	191.9	Tokuhiro et al., 2009
*S. cerevisiae* EGY48	Overexpression of *HMG2* with a K6R stabilizing mutation in Hmg2p, an HMGR isoenzyme	18.3	ND	Mantzouridou and Tsimidou, 2010
*S. cerevisiae* BY4741	Overexpression of *tHMG1* and *POS5* with mitochondrial presequence	58.6 ± 1.43	28.4 ± 1.08	Paramasivan and Mutturi, 2017
	Overexpression of *tHMG1* and *POS5* without mitochondrial presequence	33.0 ± 2.96	46.0 ± 4.08	
*S. cerevisiae* BY4741	Overexpression of *ERG9* and *POS5* without mitochondrial presequence	ND	85	Zhuang and Chappell, 2015
	Overexpression of *ERG9*and *tHMGR*, insertion mutation in *ERG1*	ND	270	
*S. cerevisiae* AH22	Overexpression of *tHMG1* under constitutive promoter	ND	ND	Polakowski et al., 1998
*S. cerevisiae* BY4742-TRP	Overexpression of *tHMG1, LYS2*	ND	150.9	Dai et al., 2014
	Overexpression of *tHMG1, LYS2, ERG9, ERG1*, expression of *bAS* (b-amyrin synthase) from *Glycyrrhiza glabra*	ND	183.4	
*S. cerevisiae* SR7	Co-expression of*tHMG1* and *ERG10* gene in xylose-rich medium	ND	532	Kwak et al., 2017
*S. cerevisiae* Y2805	Overexpression of *tHMG1*, expression of *ispA*	ND	400 ± 45	Han et al., 2018
	Overexpression of *tHMG1*, expression of *ispA*, fed-batch fermentation	ND	1026 ± 37	
	Overexpression of *tHMG1*, expression of *ispA*, fed-batch fermentation with supplementation of terbinafine	ND	2011 ± 75	
*S. cerevisiae* BY4742	Overexpression of *tHMGR* and *upc2.1* (a mutated regulatory factor that induces sterol biosynthetic gene)	ND	78	Dai et al., 2012
*S. cerevisiae* INVSc1	Overexpression of *tHMG1, IDI1* (isopentenyl diphosphate-isomerase), *ERG20* (farnesyl diphosphate synthase), and *ERG9*	ND	34	Rasool et al., 2016a
	Overexpression of *tHMG1, IDI1, ERG20*, and *ERG9*, supplementation of terbinafine	ND	119.08	
	Overexpression of *tHMG1, IDI1, ERG20, ERG9, ERG10* (encoding acetyl-CoA C-acetyltransferase)*, ERG13* (HMG-CoA synthase)*, ERG12* (mevalonate kinase)*, ERG8* (phosphomevalonate kinase), and *MVD1* (diphosphomevalonate decarboxylase)	ND	304.49	
*S. cerevisiae* INVSc1	Overexpression of squalene biosynthetic pathway using a library of 13 new constitutive promoters	ND	100	Rasool et al., 2016b
	Overexpression of squalene biosynthetic pathway using a library of 13 new constitutive promoters, supplementation of terbinafine	ND	304.16	
*S. cerevisiae* D452-2	Overexpression of *tHMG1* and *DGA1*, fed-batch fermentation in nitrogen restricted minimal media	ND	445.6	Wei et al., 2018
*E. coli* BL21(DE3)	Expression of *hopA* and *hopB* (squalene/phytoene synthases) together with *hopD* (farnesyl diphosphate synthase) from *Streptomyces peucetius*	ND	4.1	Ghimire et al., 2009
	Overexpression of *dxs* and *idi* (rate limiting enzymes), expression of *hopA* and *hopB* together with *hopD* from *Streptomyces peucetius*	ND	11.8	
*E. coli*	Expression of *hpnC, hpnD*, and *hpnE* from *Zymomonas mobilis*	ND	ND	Pan et al., 2015
	Expression of *hpnC, hpnD*, and *hpnE* from *Rhodopseudomonas palustris*	ND	ND	
*E. coli* XL1-Blue	Expression of human *SQS* (*hSQS*)	ND	4.2	Katabami et al., 2015
	Co-expression of *hSQS*, chimeric mevalonate pathway containing *tHMGR, ERG13* (hydroxymethylglutaryl-CoA synthase), *ERG12* (mevalonate kinase), *ERG8* (phosphomevalonate kinase) and *MVD1* (mevalonate diphosphate decarboxylase) from *S. cerevisiae*, overexpression of *atoB* (acetyl-CoA acetyltransferase), *idi* (isoprenyl diphosphate isomerise) and *ispA* (farnesyl diphosphate synthase)	54	230	
	Co-expression of *Thermosynechococcus elongatus SQS* (*tSQS*), chimeric mevalonate pathway containing *tHMGR, ERG13, ERG12, ERG8*, and *MVD1* from *S. cerevisiae*, overexpression of *atoB, idi*, and *ispA*	55	150	
*E. coli* XL1-Blue	Expression of *hSQS*	ND	2.7 mg/L	Furubayashi et al., 2014a
*Synechocystis* sp. PCC 6803	Disabling *shc* (squalene hopene cyclase)	ND	0.67 /OD_750_	Englund et al., 2014
*Synechococcuselongatus* PCC 7942	Overexpression of *dxs* and *idi*, expression of *ispA* from *E. coli*	ND	4.98 ± 0.90 /OD_730_	Choi et al., 2016
*S. elongatus* PCC 7942	Expression of CpcB1-SQS protein	ND	7.16 ± 0.05/OD_730_	Choi et al., 2017
	Increased gene dosage of CpcB1-SQS by strong endogenous *cpcB1* promoter	ND	11.98 ± 0.49 /OD_730_	
*Rhodopseudomonas palustris* TIE-1	Disabling *shc*	3.8	ND	Xu et al., 2016
	Disabling *shc* gene, co-expression of *crtE* and *hpnD*	12.6	ND	
	Disabling *shc* gene, co-expression of *crtE* and *hpnD*, overexpression of *dxs*	15.8	ND	
*Yarrowia lipolytica*	Overexpression of *acs* (from *Salmonella enterica*), ylACL1 (encodes acetyl-CoA synthase), and *ylHMG1*	3.3	ND	Huang et al., 2018
	Overexpression of *acs* (from *Salmonella enterica*), ylACL1 (encodes acetyl-CoA synthase), and *ylHMG1*, addition of 20mM sodium acetate	7	ND	
	Overexpression of *acs* (from *Salmonella enterica*), ylACL1 (encodes acetyl-CoA synthase), and *ylHMG1*, addition of 10mM citrate	10	ND	
*Chlamydomonas reinhardtii* C-9	Overexpression of *CrSQS*, knocked down *CrSQE*.	0.9-1.1	ND	Kajikawa et al., 2015

*HMGR, HMG-CoA reductase; tHMG1, truncated HMG1; tHMGR, truncated Hydroxymethylglutaryl-CoA reductase*.

From Table 1, all squalene values associated with Ryan et al. (2006) work (brazil nut, pecan, pistachio, cashew, and pine nut) have been deleted as the authors consider the values in their original article to be impractical. Also, the concentration of squalene in rape seed and wine lees were mentioned as 17 and 60 mg/100 g DCW, respectively, which has been corrected. For rape seed it is 43.7 mg/100 g and for wine lees it is 6,000 mg/100 g.

In Table 3, some titer values (Mantzouridou et al., 2009; Chen et al., 2010; Fan et al., 2010) were mistakenly stated incorrectly following errors while converting units. In the case of Mantzouridou et al. (2009), the titers were incorrectly provided as “2.96^*^10^**3**^ and 3.12^*^10^**3**^ g/L” while they should be “2.96^*^10^**−3**^ and 3.12^*^10^**−3**^” g/L, respectively. As for Fan et al. (2010), the corrected titer value is “2.21^*^10^**−3**^” instead of “**21.2 g/L**.” Additionally, the biomass weight was earlier stated as “**No Data (ND)**” but it was later found to be “**0.37** mg/g” dry cell weight (DCW) when the glucose concentration was 30 g/L. Lastly, for Chen et al. (2010), the titer was incorrectly provided as “**5.90 g/L**” while it is “5.90^*^10^**−3**^ g/L.” The work of Kaya et al. (2011) has been cited again in Table 3 (cited priorly in Table 2) pertaining to its fermentation parameter optimization.

As for Table 4, the squalene biomass and yield values under Paramasivan and Mutturi's work 2017 have been corrected. Upon correction, the squalene biomass and yield in presence and absence of mitochondrial presequence have been labeled separately. The squalene biomass with the mitochondrial sequence happens to be 58.6 ± 1.43 mg/g DCW, while the yield is 28.4 ± 1.08 mg/L. Squalene biomass and yield without the mitochondrial presequence is 33.0 ± 2.96 mg/g DCW and 46.0 ± 4.08 mg/L, respectively.

The corrected Tables 1, 3, and 4 appear below.

Additionally, there were errors in the text. Following the deletion of Ryan et al.'s work from Table 1, paragraph 2 under “Squalene From Plants” has been reformed as follows:

“Rice bran, a co-product of the rice milling process also contains a good amount (318.9–320 mg/100 g) of squalene (Rukmini and Raghuram, 1991; Pokkanta et al., 2019). Palm oil has just 20–50 mg/100 g of squalene (Goh et al., 1985; Lau et al., 2005) but because of its large-scale production, it can be considered as an acceptable source of the squalene overall. Apart from this, avocado (34–37 mg/100 g squalene) (Gutfinger and Letan, 1974) has also been reported to contain a meager amount of squalene. Some nuts also contain small amounts of squalene, including brazil nut (145.8 mg/100 g) (Derewiaka et al., 2014), peanut (27.4–132.9 mg/100 g) (Frega et al., 1992; Tuberoso et al., 2007; Pokkanta et al., 2019), hazelnut (9.3–39.2 mg/100 g) (Frega et al., 1992; Bada et al., 2004; Derewiaka et al., 2014), macadamia (7.2–38.3 mg/100 g) (Maguire et al., 2004; Wall, 2010; Derewiaka et al., 2014), pecan (20.8–29.8 mg/100 g) (Derewiaka et al., 2014; Fernandes et al., 2017), pistachio (5.5–22.6 mg/100 g) (Derewiaka et al., 2014; Salvo et al., 2017), cashew (11.6 mg/100 g) (Derewiaka et al., 2014), almond (1.3–9.6 mg/100 g) (Liu et al., 1976; Fernandes et al., 2017), and walnut (0.09–0.94 mg/100 g).”

Following the correction in the concentration of squalene in rape seed in Table 1, the value of the same in the manuscript (“Squalene From Plants”; paragraph 3) been corrected as **43.7** mg of squalene per 100 gm DCW.

Additionally, in paragraph 4, the following correction has been made: “Similarly, soybean, sunflower, canola, and palm fatty acid distillates encompass about **18–35**, 43–45, 30–35, and 2–13 g/kg of squalene, respectively (Naziri et al., 2011b)” has been changed to “Similarly, soybean, sunflower, canola, and palm fatty acid distillates encompass about **18–55**, 43–45, 30–35, and 2–13 g/kg of squalene, respectively (Dumont and Narine, 2007; Naziri et al., 2011b; Naz et al., 2014).”

Two corrections have been made in “**Fermentation Optimization for Squalene Production**.” In paragraph 2, “The maximum squalene production was noted to be 2.97 ± 0.12 and 3.13 ± 0.11 mg/L, whilst productivity of **0.10** **±** **0.04** and **0.16** **±** **0.05** mg/L/h was gained for *S. cerevisiae* BY4741 and EGY48, respectively (Mantzouridou et al., 2009).” has been changed to “The maximum squalene production was noted to be 2.97 ± 0.12 and 3.13 ± 0.11 mg/L, whilst productivity of **0.10** and **0.16** mg/L/h was gained for *S. cerevisiae* BY4741 and EGY48, respectively (Mantzouridou et al., 2009).”.

In paragraph 3, It was stated “In an experiment, squalene content was lifted to **21.2 g/L** with a glucose concentration of 60 g/L.” while it should be “In an experiment, squalene content was lifted to **2.21 mg/L** with a glucose concentration of 30 g/L.”

In “**Engineering** ***Saccharomyces cerevisiae* for Squalene Production**”, paragraph 2, “Additionally, this has been further improved to **250** mg/L by expressing the truncated *HMGR* (*tHMGR*) gene (Zhuang and Chappell, 2015).” has been changed to “Additionally, this has been further improved to **270** mg/L by expressing the truncated *HMGR* (*tHMGR*) gene (Zhuang and Chappell, 2015)”. Additionally, in paragraph 3, “Eventually, the complete biosynthetic pathway for squalene was overexpressed and that obtained a yield reaching as high as **304.09** mg/L (Rasool et al., 2016a).” has been changed to “Eventually, the complete biosynthetic pathway for squalene was overexpressed and that obtained a yield reaching as high as **304.49** mg/L (Rasool et al., 2016a).”

The authors apologize for these errors and state that this does not change the scientific conclusions of the article in any way. The original article has been updated.
